# KaBOB: ontology-based semantic integration of biomedical databases

**DOI:** 10.1186/s12859-015-0559-3

**Published:** 2015-04-23

**Authors:** Kevin M Livingston, Michael Bada, William A Baumgartner, Lawrence E Hunter

**Affiliations:** Computational Bioscience Program, University of Colorado Anschutz Medical Campus, Aurora, CO USA

**Keywords:** Knowledge representation and reasoning, Semantic data integration, Biomedical, Databases, Open biomedical ontologies, Semantic web, OWL, RDF

## Abstract

**Background:**

The ability to query many independent biological databases using a common ontology-based semantic model would facilitate deeper integration and more effective utilization of these diverse and rapidly growing resources. Despite ongoing work moving toward shared data formats and linked identifiers, significant problems persist in semantic data integration in order to establish shared identity and shared meaning across heterogeneous biomedical data sources.

**Results:**

We present five processes for semantic data integration that, when applied collectively, solve seven key problems. These processes include making explicit the differences between biomedical concepts and database records, aggregating sets of identifiers denoting the same biomedical concepts across data sources, and using declaratively represented forward-chaining rules to take information that is variably represented in source databases and integrating it into a consistent biomedical representation. We demonstrate these processes and solutions by presenting KaBOB (the Knowledge Base Of Biomedicine), a knowledge base of semantically integrated data from 18 prominent biomedical databases using common representations grounded in Open Biomedical Ontologies. An instance of KaBOB with data about humans and seven major model organisms can be built using on the order of 500 million RDF triples. All source code for building KaBOB is available under an open-source license.

**Conclusions:**

KaBOB is an integrated knowledge base of biomedical data representationally based in prominent, actively maintained Open Biomedical Ontologies, thus enabling queries of the underlying data in terms of biomedical concepts (*e.g.*, genes and gene products, interactions and processes) rather than features of source-specific data schemas or file formats. KaBOB resolves many of the issues that routinely plague biomedical researchers intending to work with data from multiple data sources and provides a platform for ongoing data integration and development and for formal reasoning over a wealth of integrated biomedical data.

**Electronic supplementary material:**

The online version of this article (doi:10.1186/s12859-015-0559-3) contains supplementary material, which is available to authorized users.

## Background

The depth and breadth of curated knowledge in molecular biomedicine is staggering. The 2015 Nucleic Acids Research peer-reviewed compilation of molecular biomedical databases lists 1,621 databases [1], many of which hold millions of detailed records about biomedically significant entities. Much contemporary biomedical research depends on broad and unbiased assays at genomic scale. Interpretation of the results of such assays, which generally implicate hundreds or even thousands of relevant gene products (or polymorphisms, etc.) in the context of what is already known is particularly challenging, and existing approaches are clearly inadequate to address this challenge and others. The aggregate consequences of this failure to capitalize on existing knowledge for the interpretation of genome-scale experimental results is a substantial reduction in the efficiency of the biomedical research enterprise writ large, delaying the development of both key insights and new therapies. The ability to query many independent biological databases using a common, community-driven semantic model would facilitate deeper integration and more effective utilization of these diverse and rapidly growing resources.

Attempts to access and integrate data from multiple public biomedical databases are often plagued with issues stemming from heterogeneous database schemas, idiosyncratic file formats, data redundancy, numerous independent identifiers, and differing curation standards and practices. While researchers’ decisions about which databases and which data to use should be based on their task and on biomedical criteria, they are often based instead on logistical criteria such as the underlying database representations, the ease or difficulty of accessing the data, and the ability to integrate a given data set with others. Researchers need an environment not only in which these data are readily accessible but also where they can ask queries that are biological in nature and unencumbered from the underlying shape or format of the data.

Goble and Stevens [2] have written of several serious issues in need of addressing for biomedical data integration, including the need for shared identities and semantics, the need to use existing standards where available, and balancing data collection with data use; they state that these problems have led to a current “loose federation of bio-nations”. While work to integrate various data is progressing, Good and Wilkinson point out that we are seeing “‘semantic creep’—timid, piecemeal and *ad hoc* adoption of parts of standards” [3]. Linking data across resources is necessary for building integrated systems; however, linking the data without understanding the semantics of those links merely generates more data [4]. Furthermore, any data integration must be able to support multiple modes of reasoning that can deal with the fact that integrated data are likely to have noise and errors [5].

While many domains have developed standard file formats for the consistent sharing of data, these formats are generally domain- or task- specific, making them difficult to integrate with one another. Other existing non-ontologically grounded approaches to data integration include maintaining cross references that point to related identifiers and records in other sources but often conflate semantics, *e.g.*, by linking a protein record to a gene record (such as some mappings provided in large curated databases). Other linked data approaches typically have weak semantic abstractions that do not map to a single common biomedical model and do not unambiguously assert which biomedical entities are semantically identical across data sources. This is a serious hindrance, as Goble and Stevens have posited: ′The failure to address identity will be the most likely obstacle that will stop mashups, or any other technology or strategy, becoming an effective integration mechanism” [2]. Ontology-based approaches to data integration thus far have been either small or focused on specific domains or tasks. Larger semantic integrations have not provided declarative representations of mappings, or use non-standard semantic models. Despite decades of effort, the goal of integrating diverse data into a common biomedical model remains elusive.

We have put together a set of five methods, some novel and some that build on prior work of others, that overcome problems commonly encountered when attempting to semantically integrate information from multiple data sources, When applied collectively these methods resolve seven key problems. (1) varying identifiers used across data sources to refer to the same concepts; (2) differing file formats using different lexicalizations and interpretations of identifiers; (3) conflation of informational entities (*e.g.*, identifiers and records) with biomedical concepts (*e.g.*, genes and gene products, processes and interactions); (4) the use of varying non-ontologically grounded semantic models; (5) errors and inconsistency among source data; (6) instability of identifiers and URIs over time in integrated resources; and (7) difficulty in tracing and reporting provenance of integrated data.

We demonstrate these solutions by presenting KaBOB (the Knowledge Base of Biomedicine), a system that integrates 18 sources of biomedical data using 14 prominent Open Biomedical Ontologies (OBOs) as a foundation and vocabulary for modeling, thus facilitating interaction with the wealth of existing data and tools that already rely on these OBOs. In KaBOB, identity across data sources is maintained through the generation of a single biomedical entity for each set of data-source-specific identifiers each referring to the entity. These entities, along with the OBO concepts, function as the building blocks for the common biomedical representations, which can be simultaneously modeled and thus queried at multiple levels of abstraction. KaBOB maintains a clear distinction between source data and biomedical concepts and represents both explicitly. Users need only understand the common OBO-based representations to interact with data from all of the integrated sources that have been mapped into the biomedical representation, rather than having to know each relevant source’s specific modeling and the similarities and differences among each data model; however, for data that have not been mapped to biomedical concepts, the source data are also available for querying over a common informational metamodel. KaBOB uses declaratively represented forward-chaining rules to map from the source data to biomedical concepts, and the explicit representation of both the source data and the rules together provide transparent and computable provenance for every concept and assertion. By resolving many of the issues that routinely plague biomedical researchers intending to work with data from multiple data sources simultaneously, KaBOB provides a platform for ongoing data integration and development and for formal reasoning over a wealth of integrated biomedical data.

## Methods

Biomedical data sources tend to use various idiosyncratic data models that often do not integrate well with each other, and no common model exists across them. Consequently, there is no immediately straightforward way to combine their data. To build KaBOB we tackle the problem of mapping data source contents to a common biomedical model incrementally. First we explicitly represent the contents of the data sources as informational constructs (*e.g.*, records, identifiers), then apply declarative rules to create representations of the biomedical entities they denote, grounded in a common model that is based on the OBOs. This division of information entities and biomedical concepts is one of the fundamental ideas underlying KaBOB. It greatly simplifies the manner in which biomedical representations are created and edited, as well as provides several additional advantages: for example, functioning as provenance. Furthermore it easily allows multiple representations of the source data to be generated, for example at differing levels of granularity or generalization, all still coherent with the overall model. Since each step in KaBOB construction is separate and incremental they can be developed and debugged independently, by teams of developers with differing skill sets. For example, the KaBOB approach separates computational systems design decisions (*e.g.*, how to read multiple file formats) from ontological decisions related to biomedical model representation, processes that are intermingled in other approaches.

We use five methodological steps to solve major problems commonly encountered in semantic data integration. (1) Data source records are explicitly represented using a common informational model. (2) References to identifiers in these records are canonicalized. (3) Identifier mappings across data sources are used to derive sets of IDs that are intended to refer to the same biomedical concept, and (4) these identifier sets and a corresponding biomedical entity for each are explicitly represented. Finally, (5) forward-chaining rules are used to produce representations that are grounded in common biomedical models in the form of prominent OBOs that build upon the unified biomedical entities. This modeling is done in such a way as to avoid conflicts in the event of inconsistent underlying data. Each step is discussed individually, in the context of applying them collectively to produce KaBOB.

KaBOB has three major subdivisions of representation: (1) an imported collection of prominent OBOs that serve as the representational foundation for the rest of the knowledge base; (2) the representation of source database records, schemas, and identifiers, modeled as instances of information content entities (extended from the Information Artifact Ontology [6]), collectively referred to as the ICE content of the knowledge base,; and (3) the representation of biomedical concepts such as biological processes and interactions, diseases and phenotypes, and genes, gene products, and other types of biological sequences (extended from OBOs such as the Gene Ontology [7] and the Sequence Ontology [8]), collectively referred to as the BIO content of the knowledge base.

**Figure 1:**
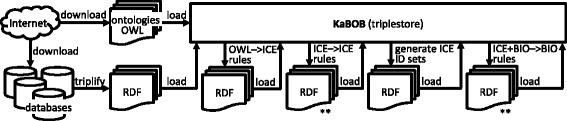
KaBOB Construction. Depicts the incremental construction of KaBOB. Labeled arrows represent processes that flow from inputs to outputs. Construction starts with downloading files and flows through translating them into RDF and then iteratively querying and producing more RDF. Steps marked with ** involve multiple sets of rules being run and their output loaded in sequence.

Figure 1 depicts how KaBOB is constructed. KaBOB, initially an empty triplestore, is built up incrementally. First, ontologies are downloaded and then loaded directly into the triplestore. Database source files are downloaded and converted to RDF; the resultant RDF triples are loaded into the ICE section of KaBOB. Forward-chaining rules (OWL- > ICE) generate ICE identifiers for each of the biomedical concepts in the ontologies. (These additional identifiers are required since in our representation records will only contain URIs for identifiers not URIs for biomedical concepts themselves, making the ICE-BIO distinction unambiguous). The second set of forward-chaining rules generates identity links between ICE identifiers, specifically, assertions of skos:exactMatch links between identifiers denoting the same biomedical concepts. The next step instantiates an ID set in the ICE side of KaBOB corresponding to each unique biomedical concept. Each biomedical concept is then explicitly represented in the BIO section; for example, a gene entity on the BIO side is created for each such set of gene IDs. (This is the first real connection from the ICE section to the BIO section). More forward-chaining rules are then used to create (on the BIO side) other biomedical concepts and assertions referred to within the data source records, *e.g.*, interaction events with protein participants from protein-protein interaction database records, processes with participating entities from Gene Ontology annotations, and links from drugs to genes or gene products from drug-related data sources. When the rules have finished running and their output has been loaded into the BIO side, KaBOB is ready to be queried and used. Each of the steps required to build an instance of a KaBOB knowledge base is discussed in greater detail in the following subsections. A detailed list of the steps used to build KaBOB is provided in Additional file 1.

### Sources

The initial release of KaBOB has been designed to include a wide variety of biomedical data from a number of prominent publicly available sources. These data range from attributes of core biomedical entities (*e.g.*, genes, proteins) to interactions between these entities (*e.g.*, protein-protein interactions, gene/transcription-factor interactions) to biological functions attributed to the entities (*e.g.*, Gene Ontology annotations). KaBOB is designed to be extensible; as such, the list of data sources should not be considered exhaustive or limiting. New data sources are being added as needed to accomplish specific reasoning and querying tasks.

KaBOB currently imports the following 14 ontologies:

1. Basic Formal Ontology (BFO) [9]
2. BRENDA Tissue / Enzyme Source (BTO) [10]
3. Chemical Entities of Biological Interest (ChEBI) [11]
4. Cell Type Ontology (CL) [12]
5. Gene Ontology including biological process, molecular function, and cellular component (GO) [7]
6. Information Artifact Ontology (IAO) [6]
7. Protein-Protein Interaction Ontology (MI) [13]
8. Mammalian Phenotype Ontology (MP) [14]
9. NCBI Taxonomy [15]
10. Ontology for Biomedical Investigation (OBI) [16]
11. Protein Modification (MOD) [17]
12. Protein Ontology (PR) [18]
13. Relation Ontology (RO) [19]
14. Sequence Ontology (SO) [8]

KaBOB currently imports data from the following 18 data sources:

1. Database of Interacting Proteins (DIP) [20]
2. DrugBank [21]
3. Genetic Association Database (GAD) [22]
4. UniProt Gene Ontology Annotation (GOA) [23]
5. HUGO Gene Nomenclature Committee (HGNC) [24]
6. HomoloGene [25]
7. Human Protein Reference Database (HPRD) [26]
8. InterPro [27]
9. iRefWeb [28]
10. Mouse Genome Informatics (MGI) [29]
11. miRBase [30]
12. NCBI Gene [31]
13. Online Mendelian Inheritance in Man (OMIM) [32]
14. PharmGKB [33]
15. Reactome [34]
16. Rat Genome Database (RGD) [35]
17. Transfac [36]
18. UniProt [37]

The utility of KaBOB is predicated not only on the knowledge it contains but also by how up-to-date this knowledge is. We have eased this knowledge acquisition process by automatically downloading individual data sources from their corresponding locations on the Internet and constructing file parsers that can detect changes in data source file formats and report back if the parsers need to be updated. Logs are kept for every download recording the date and location of each source file, and logs are generated for every file parse recording warnings and errors that need to be addressed. The entire process from download to final knowledge base creation can be accomplished in under 2 days, allowing KaBOB to be updated at the same frequency as the major data sources it depends on. Changes to the format of the data sources can require modifications to the file parsers, which is relatively straightforward, but does require some time, typically about a day. Efficient storage of and access to historic copies of all ICE data is being investigated (see Future Work).

### Database record representation

**Figure 2:**
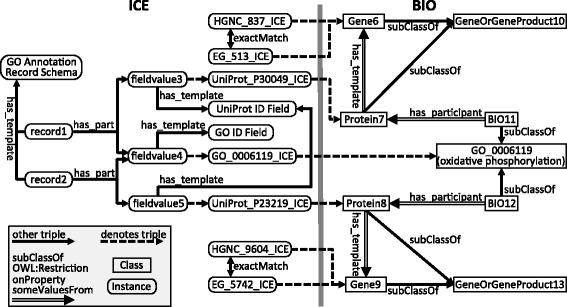
Example ICE Records and corresponding BIO Concepts. Depicts an excerpt of the knowledge representation in KaBOB. Ovals are used to depict instances, and rectangles classes. Single line arrows represent triples and point from their subject to their object and are labeled with their property. The iao:denotes links that cross from the ICE to the BIO side are emphasized with dashed arrows. The double arrows are shorthand for representing an owl:Restriction on the given property with some values from the object value. This figure depicts two GO annotation records that are then converted to biomedical concepts using the same rule (rule not depicted). Additionally sets of gene identifiers are also depicted that denote their corresponding gene concept. On the BIO side the relations between genes, proteins, and gene or gene product aggregate classes are also shown. Other than the records and their field values, generated by the file parsers, all other links are the output of applying rules.

All database content is directly modeled as information content entities (ICEs) as defined in the Information Artifact Ontology, one of the Open Biomedical Ontologies [6]. An initial model for the database record representation is discussed in detail in a previous publication [38]. Briefly, each database, schema, record, field, and field value is modeled as an ICE. The obo:has_part relation is used to connect record ICEs to their corresponding field value ICEs and database schema ICEs to component field ICEs, while the kiao:has_template relation is used to link record ICEs to their corresponding schema ICEs and field value ICEs to corresponding field ICEs. The simplicity and generality of this record representation permits its use toward the many different data sources being incorporated into KaBOB, regardless of the underlying file format (*e.g.*, CSV, XML). An example of this representation can be seen in the ICE panel (left side) of Figure 2. This figure in part depicts two simplified Gene Ontology annotation records (record1 and record2), which are each connected to two field values, one a UniProt ID and one a GO ID. Note that since both source records use the same GO ID, these two record ICEs use the same field value ICE instance (fieldvalue4); each such reuse of a field value ICE instance keeps the three triples required to define it from being redundantly represented in the knowledge base. At the scale of the complete knowledge base, this reuse of field values results in a large reduction in the total number of triples required to represent the ICEs. For the NCBI Gene Info file this amounts to an approximate 44% reduction in the number of required triples. Each field value is linked to an associated template that indicates the field for which it serves as a value (*e.g.*, in a CSV file this would be the column name), since fieldvalue3 and vieldvalue5 are values of the same field, they point to the same field ICE instance. Each field is connected to its corresponding value.

The record representation used by KaBOB differs from the previously published representation of Bada *et al.* [38] by being more record-centric. We now allow records to share structure with other records, for example to share field value instances such as in the aforementioned example, greatly reducing the number of triples required. A minor difference between the current and former representations is that record ICE instances are now linked via obo:has_part to field value ICE instances in KaBOB, whereas field value ICE instances were linked via obo:part_of to record ICE instances in the former representation of data source records.

The use of the SHA-1 hash enables the ICE URIs to be deterministically generated and reused whenever a field with the identical value is encountered without the need to keep track of field values previously encountered during construction. This also provides consistency across KaBOB instance builds, which aids in checking for differences and debugging. For example, when specifying the URI for an NCBI Gene database taxonomy field with value 9606, our optimization uses the SHA-1 hash function [39] over the field value (“NCBITaxon:9606” in this case) and incorporates the resultant hash value into the URI, *e.g.*, http://kabob.ucdenver.edu/iao/eg/F_EntrezGeneInfoFileData_taxonID_0bUKStY0wb-D665TtwobPzrc1xI .

### Canonicalization of identifiers in records

While we attempt to generate records that are as faithful to the source representation as possible, we do modify the records by transforming identifier strings to canonicalized URIs. For example, the NCBI Gene identifier for the human ATP5D gene is rendered in source records as “EG513”, “EG_513”, “EG:513”, “513” (in a field designated to contain identifiers from the NCBI Gene database), etc., all of which would be canonicalized to the same URI for that NCBI Gene identifier, *i.e.*, http://kabob.ucdenver.edu/iao/eg/EG_513_ICE .

### Forward-chaining rules

Assertions in KaBOB are generated using a series of declaratively represented forward-chaining rules. These rules take information that is variably encoded among the records of the disparate source databases and create RDF assertions uniformly represented in terms of prominent OBOs. There are several batches of forward-chaining rules, as depicted in Figure 1, and the triples generated by the rules are saved in compressed N-Triple files and then loaded into the KaBOB triplestore.

The following is an example rule that corresponds to the example in Figure 2, where some of the symbol names have been simplified for readability. (A full version of this rule is provided in Additional file 2). This rule queries record ICEs imported from the Gene Ontology Annotation database [23] to retrieve UniProt ID ICEs and GO biological process ID ICEs. It uses the triples linking ID ICEs to biomedical concepts (via the iao:denotes relation) to retrieve the corresponding protein class for the protein ID and the corresponding biological process for the GO ID using the following graph pattern.

The rule then uses the following template (depicted here in Turtle RDF format) to construct additional triples.

These five triples specify a new process class, formally defined as a subclass of the given GO process with a restriction that the given protein is a participant in the process. This new class captures the “all-some” semantics of a GO biological process annotation, describing the subclass of the biological process such that for each instance of that subclass there exists some instance of the specified protein that participates in that process [40]. It uses existing known values for ?protein and ?goProcess retrieved as bindings by the body of the forward-chaining rule, and it creates new URIs for the restriction ?participant and the dynamically generated ?newProcess class. Figure 2 shows an example of the input and output to such a rule. The rule would be run for record1 and record2, it would get the protein ID value of the UniProt ID field, and the GO ID value of the GO ID field in each of these records, then get the denoted concepts (*i.e.*, the corresponding protein and biological process classes) by following the dotted lines into the BIO representation (Figure 2, BIO panel, right side). Then, new classes of biomedical concepts would be dynamically generated (BIO11 and BIO12) and connections made to existing biomedical concepts. In this case, each would be made a subclass of the GO process specified in the original GO annotations (oxidative phosphorylation) and a subclass of a restriction specifying the given protein as a participant in the process.

The rules are represented using a domain-specific language written using Clojure s-expressions. It is an extension of the pattern language provided in the open-source KR Clojure library [41]. The rules are applied using a straightforward implementation of a forward chainer in Clojure. The rules could also be serialized to other formats. SWRL [42] is an obvious potential target; however, SWRL rules cannot have unbound variables in the head, thus blocking reification, which is needed for many (though not all) rules. The rules could be realized as SPARQL CONSTRUCT queries as well. This avenue has not been explored in great detail, as SPARQL 1.1 was still in its infancy and access to the functions necessary to reify new entities was extremely limited at the time of the start of the KaBOB project. This can be reinvestigated as future work, along with providing RIF (Rule Interchange Format) [43] export and import of rules. A complete example of a rule and more discussion is provided in Additional file 2.

### Identifiers and identifier sets

Since different data sources use different identifiers to refer to the same given concept, aggregating data across sources requires managing sets of identifiers that are intended to refer to the same concepts. In KaBOB, identifier sets are built incrementally. First, mappings between identifiers mentioned in the underlying data sources are extracted and explicitly represented. Then, unified identifier sets are derived from all relevant extracted mappings. A corresponding biomedical entity is created for each identifier set, *e.g.*, a protein for a given set of protein IDs that refer to this protein. We do this work in a series of stages, allowing the output of each stage to be used on its own as well as enabling the results to be easily inspected and debugged, as identifier mappings in individual data sources can be idiosyncratic and occasionally incorrect. These data further serves as provenance for how the biomedical entities are created.

Identifier mapping idiosyncrasies can arise from information in data sources being represented at varying levels of abstractions and granularity, requiring additional effort to understand and disambiguate the identifier mappings that they provide. For example, data sources, such as NCBI Gene Info [31], often use a field called “database cross-reference” (or “dbXref”) that may have mappings to different types of things, *e.g.*, related drugs, diseases, pathways. Care must be taken when navigating these fields to prevent links from being constructed between identifiers that are not actually semantically exact matches, *e.g.*, between drugs and diseases, or between genes and proteins. In order to be able to sift through the semantic ambiguities in cross-referencing fields, a step was introduced to help identify the cross-referencing intent. Identifying the intent of a cross-reference field requires knowing something about what a given identifier will ultimately refer to; however, at this stage in KaBOB construction entities are not fully represented. This circular logic was overcome by bootstrapping the KaBOB construction process with simple type information of what will be the ultimate resulting entities. Rules were created to generate this information and break the cycle.

These entity typing rules connect IDs from data sources to corresponding biomedical classes (subclasses of which will be ultimately created); for example, all NCBI Gene IDs are asserted to refer to types of DNA (pointing to the DNA class of the OBO Sequence Ontology) using kiao:denotesSubClassOf, a macrorelation for the property chain of iao:denotes and rdfs:subClassOf. (That is, a kiao:denotesSubClassOf assertion of the form X kiao:denotesSubClassOf Z entails the two triples X iao:denotes Y and Y rdfs:subClassOf Z.). After these denotesSubClassOf assertions are created, a second set of ICE-to-ICE rules use these assertions to extract only those mappings between ICE IDs from the various data sources (*e.g.*, the UniProt ID mappings file [37]) that denote semantically identical entities. For example, so as to extract only the mapped IDs stored in the dbXref field of a given NCBI Gene Info record that are denotationally semantically equivalent (*i.e.*, that denote the same entity as that denoted by the NCBI Gene ID), the executed rules pick out only those IDs that kiao:denotesSubClassOf SO:DNA, as the entities denoted by the IDs that do not satisfy this criterion are not DNA sequences and are therefore unlikely to be the same entity denoted by the NCBI Gene ID. Filtering mapped IDs by making use of our explicit linkages of ID types to types of biomedical concepts is a pragmatic solution that is both effective and efficient. The output of these identifier-mapping rules are skos:exactMatch links between ID ICEs.

Great care is taken to not link things across type. For example, even though the protein denoted by the identifier UniProt:P30049 (ATP synthase subunit delta, mitochondrial) is coded by the gene denoted by the identifier HGNC:837 (ATP5D), they are not linked with an skos:exactMatch relation, because they do not refer to precisely the same concept, in that the former denotes a class of proteins and the latter a class of genes. On the other hand, a skos:exactMatch link is created between HGNC:837 and EG:513, as they denote the same gene. (Proteins are connected to the genes that code for them by subsequent rules in the construction of the BIO portion of KaBOB). The kiao:denotesSubClassOf assertions are essential to sorting this out. A simplified example of this type of representation can be seen in Figure 2: On the ICE side are pairs of gene identifiers that refer to the same genes on the BIO side, *i.e.*, HGNC_837_ICE and EG_513_ICE, which both denote Gene6, and HGNC_9604_ICE and EG_5742_ICE, which both denote Gene9. Note that the gene identifiers denoting the same genes are linked to each other via the skos:exactMatch relation; however, there is no asserted relationship between corresponding protein identifiers and gene identifiers on the ICE side (*e.g.*, between UniProt_P30049_ICE and EG_513_ICE), only a relation between the denoted protein (Protein7) and corresponding gene (Gene6) on the BIO side.

After the skos:exactMatch links are created, their transitive closure is computed using the union-find algorithm [44]. The union-find algorithm is an efficient method for building a collection of disjoint (non-overlapping) sets given a list of pairs of members that are in the same set. It incrementally builds and merges sets of connected components as it streams through the list of pairs. An explicit identifier set is then created for each set of ICE identifiers with a URI based on an SHA-1 hash of the sorted members of the set. The use of a hashing function in this manner allows the identifiers to be computed consistently over time, which ensures that two KaBOB instances computed from the same sources produce the same identifiers. (Like using UUIDs, hashed URIs have no dependency on each other and can be computed in parallel, unlike sequential identifiers, *e.g.*, set1, set2, …). The computation of this transitive closure, along with constructing the initial ICE RDF, are the only parts of KaBOB construction not performed using the forward-chaining rule system. It is possible to compute this transitive closure with forward-chaining rules; however, the union-find algorithm is extremely efficient, with a running time of O(log*n), compared to potentially needing multiple passes with forward chaining to compute.

As an example of computing and creating a set of identifiers each denoting the same biomedical concept, we start with the following three triples, which specify mappings between IDs denoting the same gene.

After passing through the union-find algorithm the four identifiers in these triples are grouped into a single identifier set. The following four triples (in Turtle RDF syntax) specify this set, which is given the URI kiao:KaBOB-ID-Set-qn-3e2r15NYu8WUNe-a2BXB_nZ, based on the SHA-1 hash of its members. The set is comprised of four identifiers whose membership is the set is asserted via the kro:hasMember relation:

### Representation of biomedical concepts

More rules are used to build up biomedical representations. First, a biomedical concept is created for each identifier set, *e.g.*, a gene class corresponding to the set of ID ICEs denoting the given gene. Layers of biomedical sequence abstractions (discussed in the Representation at Multiple Levels of Abstraction subsection of the Discussion section) are added, *e.g.*, aggregate gene-or-gene-product classes and gene-or-gene-product-or-variant classes. Connections are made between corresponding genes and gene products, *e.g.*, representing that a given gene serves as the indirect template for a given protein, and connecting that protein to the corresponding gene-or-gene-product aggregate classes.

The last set of rules continue to convert information variably represented in the ICE records into unified biomedical representations in terms of relevant OBO classes. Generally, each KaBOB assertion derived from a source database requires one rule and results in at least one dynamically generated subclass to model the assertion in OWL. A rough estimate is one rule per field in the source records; however, some fields require multiple rules. For example, the gene type field in the NCBI Gene database requires one rule per possible sequence type (*e.g.*, protein-coding gene, noncoding gene, pseudogene) to accurately model the value of the field, as these values are curated by NCBI using a small custom controlled vocabulary, each term of which is uniquely mapped to a Sequence Ontology class. On the other hand, extracting other types of assertions requires looking at multiple fields in one rule. For example, extracting a drug-gene association assertion from PharmGKB requires the examination of four fields, as PharmGKB uses two fields to specify the identifiers of the interacting entities and two more fields to specify the types of these entities (*e.g.*, gene, drug).

As an example of a dynamically rule-generated biomedical concept extended from existing OBO concepts, the following nine triples (in Turtle RDF syntax) represent the interaction (obo:MI_0000) between the drug isoflurane (kbio:BIO_e7687a66889760a757dd0bdc6b12ea67) and the gene ATP5D (or one of its products or variants) (kbio:GorGPorV_BIO_8b130947230c6d1bdb2a067cba2514de), derived from the Drugbank database [21] :

The first block of triples models the OWL restriction class (kbio:R_I4hmhKlPj3PLuD__6T2QucrTfWk) representing the class of all things in which the gene ATP5D or one of its products or variants participates. The second analogously models a restriction class (kbio:R_mSPMJTgCh1qGqP7t_SNOf1KF-Kk) of all things in which isoflurane participates. The third block formally defines a class (kbio:I_IB7hiBNOSmq0zEGYg6D7NXd9kQ0) as the subclass of both of these restriction classes and an OBO interaction class, *i.e.*, the subclass of interactions such that for each of its members, there exists some isoflurane that is a participant, and there exists some ATP5D or one of its products or variants that is a participant.

A large amount of redundant generation of semantically equivalent OWL Restriction classes is avoided through the use of assertions that generate unique hashes for the restrictions. For example, the URI for the restriction kbio:R_I4hmhKlPj3PLuD__6T2QucrTfWk is generated from a SHA-1 hash of the property and object values of the triples used to define it. Reusing OWL fragments like this significantly reduces the number of triples and in doing so reduces the load on reasoners that will eventually operate over KaBOB.

Currently there are 75 rules. Depending on the complexity of the rule, and if other rules that look at similar source records or that have similar output representations exist, a new rule can be written in anywhere from minutes (if it is closer to a cut and paste) to an hour or so (if more thought and exploration is required).

### Implementation

Command-line build scripts for installations in both AllegroGraph (v4.14) [45] (a state-of-the-art commercial triplestore provided by Franz Inc.) and Virtuoso (v7) [46] (an open-source triplestore based on relational databases provided by OpenLink Software) are provided in the open-source release. We run these scripts via a Hudson server in order to monitor performance and output; however, they could be run just as easily without Hudson. While we have targeted AllegroGraph and Virtuoso, there is nothing specific about KaBOB to either. To query the triplestore, the rule engine and identifier-set creation code uses the open-source KR Clojure library, which can talk to any triplestore that speaks Sesame or Jena. At most, one small function for establishing the connection to the source triplestore would have to be extended, but more than likely speaking to a different server requires only changing the parameters for server location and authentication. The build scripts would have to be extended for additional triplestores to provide a command-line call for loading a directory of RDF files into the triple store. Porting the scripts from AllegroGraph to Virtuoso was done in about a day. The command-line scripts are written in BASH, and all other code is Java or Clojure (a Lisp dialect that runs on the JVM), both configured with Maven, allowing the code to be run anywhere the JVM can. We have done all of our work on a custom-built machine with 24 cores, 96 GB RAM, 2 TB of SSD drives LVM RAID-0 (for the OS and triplestore), and 3.6 TB of spinning disk RAID (for managing the RDF files), running Fedora v17.

## Results

Two fully functional versions of KaBOB have been built, along with one partial version. One fully functional version has been built using only human source data and another using human data plus data for seven major eukaryotic model organisms (listed along with their NCBI Taxonomy IDs): *Mus musculus* (10090), *Rattus norvegicus* (10116), *Drosophila melanogaster* (7227), *Saccharomyces cerevisiae* (4932), *Caenorhabditis elegans* (6239), *Danio rerio* (7955), and *Arabidopsis thaliana* (3702). (Data for subtaxa of these taxa also represented in the NCBI Taxonomy, *e.g.*, subspecies of mice and strains of yeast, have been included.) Ideally, a version of KaBOB would be built using data for every organism included in the data sources; however, that is out of reach of our current hardware/software configuration (see discussion in the Current Limitations and Future Work section). For the all-organism version, only the ICE records have been produced. For the two fully functional versions, all KaBOB steps are performed, from initial ICE record representation to running of all of the BIO concept generating rules. Each version can be built in approximately two days.

Data is parsed from a total of 43 different files from 18 data sources. Table 1 shows the numbers of triples and compressed file sizes for the three versions of KaBOB and for these versions in total as well as for the three primary triple subsets of imported OBOs, ICEs of original source data, and rule-generated data. All versions of KaBOB use the same 14 ontology files, which amount to 13,830,676 triples. The triple subsets of imported OBO content and RDFized original source data are the two primary sources of data to KaBOB and are shown in the two parallel paths on the left side of Figure 1. The rule-generated data comprise all other triples in KaBOB and includes all of the RDF files depicted under the KaBOB block in Figure 1. Table 2 shows the number of identifier sets in each version of KaBOB, along with the number of triples required to represent those sets, and the size of those triples in compressed N-triple format.

**Table 1:** **Size of KaBOB**

	**imported OBOs**		**ICE records**		**generated (rules and id sets)**		**KaBOB total**	
**subset**	**# triples**	**size .owl (GB)**	**# triples**	**size .nt.gzip (GB)**	**# triples**	**size .nt.gzip (GB)**	**# triples**	**size (GB)**
human only	13,830,676	1.5	144,489,737	2.0	7,615,547	0.2	165,935,960	3.6
human +7 major model organisms	13,830,676	1.5	369,027,022	4.9	34,968,305	0.7	417,826,003	7.1
all organisms	13,830,676	1.5	9,584,033,541	126	n/a	n/a	n/a	n/a

Lists the size of the various collection of RDF generated in the KaBOB build process, recorded in number of triples and size on disk. The first three major columns include the imported OBOs, the ICE records (output of the file parsers), and the generated triples (output of the rules and ID merging). The fourth column is the sum of the first three. The rows represent subsets of the KaBOB data based on organisms included. The subsets are human-only, human plus seven major model organisms (listed in the paper), and the final row is for all organisms combined. Due to the scale of the data in the final subset this data is currently incomplete.

**Table 2:** **Number of entities / ID sets**

**subset**	# **id sets**	# **id set triples**	**total RDF .nt.gz file size**
human only	336,472	952,807	14 MB
human +7 major model organisms	1,513,932	3,644,255	56 MB

List the number of entities or ID sets in each subset of KaBOB. Each ID set is the collection of identifiers from multiple data sources that are intended to denote the same biomedical concept. Number of ID sets, number of triples, and size on disk is reported.

The ability to answer complex queries that target the common OBO-based biomedical model is demonstrated by a series of example queries. An extended use case is discussed in the “Following up on GSEA results; a case study for using KaBOB” section, and several other examples are provided in Additional file 3.

This project has also produced or substantially expanded three open-source software libraries. First, this project was the primary motivation for the already released KR library for working with RDF and SPARQL in Clojure [41], which has been downloaded more than 800 times. In addition, timed with the publication of this paper is the release of two more libraries of code. The first is a Java-based project consisting of the file parsers for all of the data sources serving as input into KaBOB and code to convert the parsed files into RDF. The second project released in conjunction with this paper is the KaBOB-specific code itself. This code includes the scripts for building KaBOB, as well as the ID set merging code and all of the declarative rules.

In addition to building an integrated system that increases the value of the data from the underlying sources, we are also able to detect potential errors in the data sources and report them to their curators. Errors can be detected by querying KaBOB for assertions that should not exist, *e.g.*, a protein asserted to exist in two disjoint organismal taxa. In this way, we were able to find a mapping in DIP that erroneously equated a mouse protein to the homologous rat protein, an error that was replicated in the iRefWeb aggregation. During the identifier-merging step, we have also looked for collapses of what should be multiple entities into single entities. This has revealed errors in our own code, such as bugs in the ID canonicalization inadvertently truncating IDs and causing them to collide, as well as revealing bad mappings in the underlying data sources, including finding over 300 cases in DrugBank where multiple DrugBank records mapped to the same external identifier. Pipelined approaches to using these data sources would be susceptible to propagating such errors, as they would blindly hop through whatever mappings are being used to the target ID, potentially producing erroneous mappings. See Additional files 4 and 5 for a more detailed discussion of queries used to find errors, and a more complete explanation of the DrugBank example.

## Discussion

In this section we discuss the solutions to data integration problems that our methods overcome, and how they are manifested in KaBOB. We also provide a detailed example of querying KaBOB using SPARQL 1.1 and conclude with a discussion of limitations and future work.

### Solutions to data integration problems

The methods and knowledge representations that we have developed to integrate multiple data sources into a unified knowledge base provide solutions to seven semantic data integration problems. These solutions include: (1) distinct representations of data and biomedical concepts; (2) common biomedical representations; (3) identity resolution across data sources; (4) consistency despite potential errors or contradictions in source data; (5) the ability to represent and query data using multiple different levels of abstraction or granularity simultaneously; (6) stable and reusable URIs; and (7) traceable provenance.

### Distinct informational entities and biomedical concepts

Previous work integrating large quantities of biomedical data either use source-specific modeling and thus are actually disjoint, or do not include the portions of the source data that have not yet been represented in the common biomedical model (see discussion in Related Work). Another common problem when dealing with biomedical data is overloading the meaning of common identifiers (*e.g.*, using a UniProt identifier to simultaneously represent an identifier, a record, and a protein). KaBOB resolves these problems by clearly modeling both biomedical concepts (*e.g.*, genes and gene products, diseases and phenotypes, interactions and processes) and informational entities referring to these concepts (*e.g.*, database schemas, records, fields, field values, and identifiers) and maintaining an explicit distinction between the two categories. For example, KaBOB has distinct, explicitly represented concepts for a UniProt identifier, the corresponding UniProt record, and the class of protein that the identifier denotes and the record describes.

All of the aforementioned informational entities of the source databases are represented as information content entities on the ICE side of KaBOB. Biomedical representations are derived from these ICE data and represented on the BIO side of KaBOB. (These representations are further discussed in the next subsection). The only links that cross the demarcation between ICE and BIO representations are relations that indicate that a given informational entity on the ICE side “is about” some biomedical concept on the BIO side. There are three relations from the IAO that are used to make these connections: iao:denotes is the most frequently used one, indicating that a given ICE exists for the sole purpose of identifying a given BIO concept (*e.g.*, UniProt:Q3B891 denotes the human BRCA1 protein); iao:mentions is a weaker relation stating some part of the ICE denotes the BIO concept (*e.g.*, a given sentence mentioning the human BRCA1 protein ); and iao:is_about is the parent relation of the two, encompassing a more general sense of aboutness. The vast majority of these ICE-to-BIO links are iao:denotes assertions that are generated to link identifiers to their denoted concepts. Rules can also assert an iao:mentions link between a record from which information was retrieved to the BIO class that was dynamically created in order to represent that the class was based on information in that record. These links across the ICE-BIO divide serve as a primary source of identifying provenance data for biomedical concepts.

The clear separation and explicit representation of source data and biomedical concepts provide several distinct advantages. The availability of a common biomedical model allows queries to be written in terms of biomedical concepts, not in terms of information artifacts or database structures with which the user must become familiar, and queries over the biomedical modeling will not have to change if new source information is mapped to existing biomedical concepts and assertions. Source information that has not yet been mapped to biomedical-concept-based representations can still be queried given that it is first represented as information content entities on the ICE side. Furthermore, explicitly representing the source data makes it available as provenance for the biomedical representations, enabling queries of source evidence for given biomedical assertions (discussed further below).

### Ontology-based representations

KaBOB is modeled using representations from existing prominent biomedical ontologies created and maintained by core developers with significant community input. We rely on prominent Open Biomedical Ontologies (OBOs) as a framework. The species-specific biological sequences and their information from source databases that comprise KaBOB are placed within this framework. When necessary we extend existing ontological classes with dynamically generated composite classes (*e.g.*, a composite class representing the interaction of two given proteins, defined in terms of already explicitly represented classes for molecular interaction and for the two proteins). These composite classes are formally defined in terms of explicitly represented OBO classes, carefully maintaining the OWL “all-some” quantification among related classes. The large majority of KaBOB assertions rely on relations from the Relation Ontology (RO) [19], those used in the OBO cross-products effort [47], and natural extensions of the latter. All of the ICE representations in KaBOB are types of information content entities that we have modeled as extensions of the Information Artifact Ontology (IAO) [38].

Our use of ontological concepts and relations already explicitly represented along with the dynamically generated composite concepts formally defined in terms of these existing concepts allows us to precisely capture biomedical knowledge, including content of source databases, to arbitrary levels of complexity. Using and extending prominent OBOs as a framework enables sophisticated reasoning over the content of KaBOB. Most straightforwardly, a plethora of deductive inferences can be made based on the ontologies’ fundamental taxonomic hierarchies, non-taxonomic linkages among their classes, and the formal definitions of the active OBO cross-product efforts. This approach also opens the door for research into inductive and abductive reasoning methodologies. Furthermore, reliance on these OBOs facilitates interaction with the enormous amount of data annotated with them and with other resources making use of them. This in turn makes it easier to model and absorb source data into KaBOB as well as easier for users familiar with the community ontologies to interact with KaBOB and to formulate queries and understand their output. The representations in KaBOB are biased to be event-centric, as they are easier to represent with all-some restriction semantics. However, entity-centric representations are created for simplicity in some cases, *e.g.*, assertions linking proteins to the genes that code for them (though it would also be possible to explicitly represent the implicit transcription and translation events). Event-centric representations could be translated into entity-centric classes and assertions; however, to conform to all-some assertional semantics, this requires a somewhat more roundabout representation so that only the entity’s potential to participate in a particular event is represented as opposed to stating that all instances of a given entity necessarily participate in it.

### Identity resolved across data sources

In order to integrate data from multiple data sources it is essential to understand which identifiers across the sources fundamentally refer to the same things. This is complicated by the fact that data sources often use their own source-specific identifiers to avoid external dependencies that could cause problems in their curation efforts. Fortunately, mappings are often provided across data sources. Sometimes these mappings provide one-to-one mappings specifying identity; however, they are often provided as more convoluted sets of “related” identifiers. Great care must be taken when processing these mappings (see the previous “Identifiers and Identifier Sets” subsection).

In KaBOB, each identifier in a set, all of which denote the same biomedical concept, is directly linked to this single shared biomedical concept. We chose this approach as opposed to the alternative of modeling assertions from each data source individually on the BIO side of KaBOB and then connecting the BIO entities using owl:sameAs assertions. The alternative is more opaque and would be difficult to navigate for RDF approaches that do not make inferences over owl:sameAs assertions. Even for some systems that handle owl:sameAs the alternative approach dramatically increases the number of triples and could result in intractability. Our method of first generating an identifier set and then linking to a single corresponding biomedical concept alleviates these problems. The identifier sets are also useful in and of themselves when querying the data source records without having to travel in and out of the biomedical representations.

By resolving identity across data sources, KaBOB alleviates one of the most critical [2], time-consuming, and redundant steps [48] in integrating data from multiple sources. Systems that do not do this require users to maintain mappings across sources in every query, dramatically increasing the complexity of the query and creating ongoing maintenance problems. In KaBOB a set of trusted high-quality mappings is applied first, and then the unified entities are used to aggregate data from multiple data sources. Since the mappings are all managed and extracted using explicitly represented sets of declarative rules, it is easy to produce or maintain alternative mappings or to recompute the unified entities using a different set of trusted sources in the event a user believes that a different set of sources should be used as the basis for the mappings. This can be done by either adding rules or deactivating existing rules during KaBOB generation. The subsequent steps to building KaBOB would then proceed as normal, connecting representations to the alternate entities, and existing queries targeting the biomedical representations could still be issued without the need to be altered. Resolving the identities of data-source-specific identifiers, aggregating them into sets, and linking them to biomedical concepts they denote in common is the essential foundation that enables the querying of KaBOB in terms of the shared biomedical modeling rather than having to perform queries in terms of (often a series of) data-source-specific representations.

### Tolerance for inconsistent source data

KaBOB tolerates inconsistencies in assertions among disparate data source records on both the ICE and BIO sides of the knowledge base. On the ICE side of KaBOB, source data records are modeled as independent informational entities (specifically, records, fields, field values, and identifiers). Conflicting assertions would each be independently represented as different records. This captures the fact that one of the assertions was made in its corresponding record and the other assertion in the other record, and these two assertions may be conflicting or not if integrated. Note that our methodology also alleviates the issue of assertional inconsistencies *within* a given data source. While the semantics of conflicting assertions within most of these data sources is ambiguous, these assertions are modeled the same way as if from separate data sources, with one assertion within one record and the other assertion in the other record, all explicitly and clearly represented as informational entities.

Conflicting assertions extracted from conflicting source records can also be modeled on the BIO side without generating an inconsistency. This is enabled by generating dynamic subclasses for every assertion being modeled in the BIO portion of the knowledge base. This minimizes the places at which two assertions can conflict since each new assertion from a source database is represented as a subclass. As such each assertion is essentially in its own “world”, all contained in the KaBOB open world. These sets of subclasses can then be aggregated in multiple ways as discussed in the following section, and demonstrated in the subsequent example query. Since there is no guarantee that data from multiple sources, or even within one source, is necessarily consistent (and in fact the converse is almost a given) this is something that data integration systems, especially those using formal logics (*e.g.*, OWL) must necessarily address. Failure to do so could result in an inconsistent (and unusable) or erroneous knowledge base. By modeling representations with an extensive amount of subclassing we create an environment where the inevitable inconsistent or erroneous assertion will not ultimately result in an inconsistent knowledge base. By following this precedent when new sources are added this environment is maintained, creating a stable environment for the ongoing integration of data.

### Representation at multiple levels of abstraction

For some tasks biologists care greatly about distinctions between corresponding biological sequences, *e.g.*, genes versus gene products, reference sequences versus variants, species-specific sequences versus homologs; for other tasks, the distinctions are unimportant and so corresponding sequence types can be aggregated into more collective types. KaBOB provides a flexible knowledge model capably of representing the full spectrum of sequence type abstractions. Representations such as the collective class of genes, gene products, and variants can provide this freedom and are needed when a particular source curates a given type of data at a high level of generality. For example, with regard to a drug-gene interaction assertion in a source database, while it is possible that the given drug directly interacts with the specified gene (*i.e.*, DNA), it is much more likely that it binds to one of its products, though which product (*e.g.*, RNA, protein, or even specific protein isoform) may not be specified at this level of curation; furthermore, it may not be specified whether the interacting entity is a reference sequence or a variant. (This information may not even be known to the original researchers or the curators). We can properly model such a curated interaction by making use of a sequence abstraction class, asserting that there is an interaction between the drug and the gene or one of its products, either in the form of a reference sequence or a variant.

Certain abstractions are necessary for the current modeling being done in KaBOB and have already been explicitly modeled and used; for example, for every species-specific gene class, we have also created a corresponding abstracted class of the gene and its gene products, which subsumes the gene class and all of its products. Other abstractions could easily be created and layered on additively; for example, for species-specific gene classes, we plan on creating corresponding abstracted homology classes, *i.e.*, the class of a given species-specific gene and all of its homologs. This would allow consistent modeling of situations in which the precise identity of the homolog (*e.g.*. mouse vs. human) is ambiguous in the source information. User-defined abstractions could be layered on as well without affecting the underlying data. These abstractions can be formally defined as union classes in OWL, and the rule engine can be used to generate all of the specific classes (*e.g.*, gene-specific, protein-specific, etc.). Since these abstractions are formally defined using ontologies and generated using rules that can be traced all the way back to the records used to trigger the rules, it is completely unambiguous what is meant by any given abstraction. No implicit assumptions are made about what is being modeled. This is not true for systems that provide weaker definitions of the abstractions they employ.

In addition to sequence-based abstractions KaBOB also supports varying granularity in process-based representations as well. Multiple rules can be applied to the same underlying data to generate representations at multiple layers of abstraction. For example, it is possible to look at a pathway database that associates proteins with a pathway and initially model that as a pathway which obo:has_participant those proteins; this would model the pathway at a high level of granularity. If that pathway database also modeled the interactions that make up the pathway or the processes that inhibit or enable other processes, subsequent rules could be written to extract and model that information at finer levels of granularity. All of these rules and resultant triples can exist simultaneously and without conflict.

Representing biological sequences and processes at multiple levels of abstraction enables users to choose a level of specificity or generality for a given query; in fact, different parts of a query may be specified to operate at different levels of abstraction. Further, it requires queries to be explicit when moving between levels of abstraction and thus highlights where a query might be making non-deductive steps. This rightfully places decisions on where to make inferential leaps or how to leverage abstractions on the users and tools that need these inferences and abstractions. KaBOB makes no preferential commitment to any one level of abstraction or granularity and allows data and queries to exist at multiples levels simultaneously.

### Stable and reusable URIs

When generating assertions in KaBOB, many new entities must be explicitly represented, including informational entities such as field values and records as well as biomedical concepts such as proteins and interactions. OWL also requires the generation of anonymous identifiers such as those for dynamically generated owl:Restriction classes that are commonly modeled using RDF blank nodes. In order to provide consistency and stability over time, URIs for these entities are deterministically constructed using SHA-1 hashes of the values that functionally define these entities; for example, a specific field value of a specific field is deterministically defined by the field name and its value. Such hashing provides stable, unique, and consistent URIs for these identifiers, and by using a one-way hash function we avoid encoding data into the URIs, thus complying with the well-established guideline for URIs to be devoid of implicit meaning. The stability of the URIs over time supports debugging by consistently regenerating content the same way every time, and future work will leverage these URIs to monitor changes in data sources over time.

These hashed URIs further enable significant savings in terms of the number of triples by allowing representation to be shared in both ICE and BIO content. This is most notable in the sharing of field values and OWL restrictions; however, it also allows different databases that refer to the same biomedical concept, *e.g.*, a protein-protein interaction, to point to the same class. These connections are made without having to look up an existing URI or even know if there is an existing one, as identical URIs will be minted every time. Not having to remember or look up potentially existing URIs enables efficient parallelization as well.

Although a hashing collision is theoretically possible with this approach, SHA-1 should provide more than enough space (160 bits) to avoid it empirically. The best known theoretical attack on SHA-1 requires 2^69^ (~5.9 × 10^20^) hashes to identify a collision [49]. This is further mitigated by the fact that the hashed URIs have other components as well, *e.g.*, source-specific namespaces. A collision would have to happen within one data source or one type of thing represented in the BIO content, making an extremely unlikely event even more unlikely. There is no foreseeable need to cryptographically secure KaBOB URIs; however, if such a need arose, SHA-2 or SHA-3 hashes could be used instead at the cost of longer URIs.

### Traceable provenance

Provenance can be tracked in two primary ways in KaBOB. Most directly, concept-level provenance can be assessed via the IAO links that connect ICEs to BIO concepts, *e.g.*, an iao:denotes link between a protein ID ICE and a protein, or between a protein-protein interaction record ICE and the corresponding interaction concept in the BIO part of KaBOB. Thus, the informational source of any BIO concept is directly accessible by simply querying for the triple linking the given ICE to its denoted biomedical concept.

The second method for acquiring provenance is by running rules “backwards”. Every triple in KaBOB is from one of four sources: OWL ontologies, RDF built from the data sources, RDF output from ID set generation, or the output of rules that use the other available triples. Every triple can thus have its provenance dynamically re-derived as a set of source records (and ontology parts) and rules that created it. Note that triples in the BIO part of KaBOB can actually have multiple sources of provenance. For example, it is possible that two different rules and two different sources of drug-gene interaction information lead to the same set of triples in the biomedical representations. (It is in fact a goal of KaBOB that two sources of the same information necessarily lead to the same biomedical modeling). In addition to this provenance, records themselves often have data-source-specific provenance, *e.g*., the PubMed ID of the publication serving as the evidential basis for the creation of a given data record. These provenance data are mirrored in the record ICEs and are readily accessible for querying. Future work could include writing rules to extract and represent this type of provenance more explicitly and consistently by generating ICE-based links between the PubMed IDs and corresponding records. By directly asserting concept-level provenance assertions and by declaratively representing the transformations that generate assertions, provenance in KaBOB is completely transparent and unambiguous. This is in contrast to other existing systems that bury these transforms and the representational choices they make in procedural code (*e.g.*, a Perl script that generates triples from a data source), which is then generally opaque to users and reasoning systems. The approach taken by KaBOB affords a more direct ability to inspect and update provenance and resolve potential modeling errors when they are uncovered. Errors resulting from bad source data can also be traced back to their origins, some of which we have reported to their curators.

### Following up on GSEA results; a case study for using KaBOB

One common type of biomedical experiment is to run a high-throughput microarray analysis and compare transcription levels in case and control groups to get a list of differentially expressed genes. Researchers will then often report the results of GSEA (Gene Set Enrichment Analysis) [50] on that list. Frequently this is where the analysis ends; however there are numerous follow-up questions the researchers and readers likely have. KaBOB can be used to answer these questions.

For example, Choi *et al.* [51] were interested in changes in mitochondria in mice bred for high and low fear. They showed that the genes differentially expressed in the prefrontal cortex (PFC) were enriched for the process of oxidative phosphorylation. The authors conclude their discussion by stating that “a better understanding of the genes associated with the mitochondrial function in the PFC may provide an opportunity to identify a novel drug target for the treatment of mood and anxiety disorders.” Natural follow-up questions to these results are: “Which genes/gene products in humans, are localized to mitochondria, involved in oxidative phosphorylation, and are targets of drugs? And what are these drugs?” These questions can be readily asked and answered with KaBOB. For example, the following SPARQL 1.1 query retrieves drugs that bind to gene products that are localized to mitochondria and are involved in oxidative phosphorylation:

The query is broken into five major lettered blocks, A-E. Section A queries for the restriction of all things that have been found to localize in mitochondria, finds the corresponding localization events, and retrieves the gene products found to have localized there. For a given gene product, section B retrieves the corresponding gene-or-gene-product aggregate class, and section C queries for which of these gene-or-gene-product classes pertain to humans. Section D then returns to the gene-or-gene-product aggregate class and first retrieves all of its subclasses, including itself. Note that to effectively use the aggregate classes for every biomedical assertion one must first query for all the subclasses of the aggregate. This allows for all of the subclasses to be queried as an aggregate; if instead a variable was reused across clauses, that query would effectively be asking for one specific gene product known to satisfy all conditions (for which, in this case, none would be found). Section D continues by querying for all things in which these gene products participate, and then selecting only those that are a subclass of oxidative phosphorylation. Section E again gets a new subclass of the aggregate abstraction, then retrieves all events in which it has been found to participate, finds other participants of these events, and then makes sure that the second participant is realizing the role of (*i.e.*, acting as) a drug in that interaction. Finally, the query retrieves the names of the resulting drugs.

This query highlights some of the benefits of the KaBOB representations discussed in earlier sections. Sequence abstractions are utilized, as seen in section B of the query. The extensive use of subclassing is also evident throughout the query. For example, section A is looking for the subclass of localization, that is the subclass of all things that result in localization to the mitochondria, and then queries for the other parent classes that provide information about what is being localized. This query also highlights the fact that the query is asked only in terms of biomedical concepts (*e.g.*, genes/gene products/variants, organismal taxa, oxidative phosphorylation, mitochondria, drugs), with no informational entities (*e.g.*, names of specific databases, fields, identifiers) ever referenced.

With only PharmGKB integrated as a source of drugs and drug targets, the query above returns the two drugs adefovir dipivoxil and tenofovir. By extending the SELECT statement to include the variable ?gorgporv (the variable representing gene-or-gene-product aggregate classes) the query is modified to return gene-drug interacting pairs, instead of just the drugs. Executing this query shows that both drugs interact with the AK2 gene or its products.

After writing three rules to also integrate DrugBank into KaBOB using the same common biological representation, the query can be run without changes and the results are extended to 8 genes that collectively interact with 26 different drugs. These eight genes include two from the PFC gene list in Choi *et al.* (UQCRB, UQCRC1), two more that are mentioned in another gene list in Choi *et al.* (AK2, UQCRC2) and four that appear in neither gene list (ATP5C1, ATP5D, COX1, UQCRH).

Looking up all the drugs that interact with the PFC gene list produces 169 potential compounds. Running the query above produces a far more targeted list of 26 compounds. The five in bold do not occur in the list of 169 and thus are unique to this approach; this is due to the fact that KaBOB can identify other potentially relevant genes that were not in the experimental results that are drug targets, in this case genes involved in oxidative phosphorylation that have been found to localize in mitochondria that were not in the experimental PFC gene list.

(5S)-3-ANILINO-5-(2,4-DIFLUOROPHENYL)-5-METHYL-1,3-OXAZOLIDINE-2,4-DIONE

1-ACETYL-2-CARBOXYPIPERIDINE

2-Hexyloxy-6-Hydroxymethyl-Tetrahydro-Pyran-3,4,5-Triol

2-NONYL-4-HYDROXYQUINOLINE N-OXIDE

5-HEPTYL-6-HYDROXY-1,3-BENZOTHIAZOLE-4,7-DIONE

5-n-undecyl-6-hydroxy-4,7-dioxobenzothiazole

Adefovir Dipivoxil

AUROVERTIN B

Bis(Adenosine)-5’-Pentaphosphate

Cholic Acid

**Desflurane**

**Enflurane**

FAMOXADONE

**Halothane**

**Isoflurane**

**Methoxyflurane**

METHYL (2Z)-2-(2-{[6-(2-CYANOPHENOXY)PYRIMIDIN-4-YL]OXY}PHENYL)-3-METHOXYACRYLATE

METHYL (2Z)-3-METHOXY-2-{2-[(E)-2-PHENYLVINYL]PHENYL}ACRYLATE

Myxothiazol

N-Formylmethionine

N1-(2-AMINO-4-METHYLPENTYL)OCTAHYDRO-PYRROLO[1,2-A] PYRIMIDINE

PICEATANNOL

Quercetin

Sevoflurane

Tenofovir

UBIQUINONE-2

The purpose of this example is not to demonstrate novel biomedical findings but instead to illustrate how a complex query expressed exclusively in terms of biomedical concepts, without having to know underlying database schemas, can be formulated and issued against our integrated knowledge base. However, even a brief search of PubMed with the results from this example shows biomedical relevance of the results of the example query. For example, research has been conducted looking at the effects of isoflurane and desflurane on mitochondrial function and cognition [52].

### Current limitations and future work

The primary limitations of the KaBOB methods and knowledge model are related to issues of scale. These limitations include the size of the original data source files and the number of records they contain, limits on the number of triples a given machine or piece of software can store and query, and limits on the ability to query for triples that are entailed but not explicitly represented. Fortunately all of these issues are major and active areas of research and development in the Semantic Web community and by product vendors, so in addition to our attempts to mitigate them, it is likely that they will continue to be externally addressed.

Storage of the record RDF for the all-organism version of KaBOB takes approximately 140 GB per version. Updating KaBOB monthly and preserving legacy data would require nearly 2 TB of space per year. Part of our ongoing research is developing a methodology to require keeping only differences to historical data instead of complete copies, hopefully greatly reducing storage requirements. This is potentially feasible due to our use of a common underlying representation for records from all source databases and SHA-1 hashing to consistently name URIs that we are generating, which collectively provide a high level of consistency across versions and over time.

Early steps in the KaBOB build process are possible using the full (all-organism) data set; however, more complicated queries later in the build process were taking inordinate amounts of time on our hardware/software combination and so have been temporarily placed on hold while we work on moving to a larger machine. Triplestores tend to scale as a function of memory, and we believe an all-organism KaBOB can be computed on a larger-memory machine. Our collaborators at Franz have run some of our test queries successfully on a 384 GB RAM machine (4x what we are running). Machines with 500 GB or even 1,000 GB are becoming more common at supercomputing facilities, such as those being built at the University of Colorado, and cloud-based environments such as Amazon’s EC2 currently rent access to 244 GB machines. We will also continue to explore other potentially large but bounded subsets of the data, *e.g.*, all eukaryotes. Additionally, triplestores are continuing to improve, and the hardware needed to run them is decreasing in cost. We are operating on the order of 10 billion triples with hardware costing less than US$10,000. Distributed triplestores are an ongoing area of research, and experimental systems have been successfully fielded on 100 billion triples already.

KaBOB satisfies the data modeling desiderata for integration put forward by [2–4]. These papers also discuss what is required to have a more open and federated system of data access, but this is beyond the scope of our work. Common models for identity, semantics, and provenance are prerequisites for such a federated system, and these are some of the problems KaBOB addresses. The knowledge representations in KaBOB lay the foundation for how to integrate data using the OBOs such that data from disparate source databases are interoperable, and this work should be equally applicable to any future attempts at federation. Future work could potentially enable multiple independent end-points participating in a federated KaBOB.

Reasoning at scale can also be problematic. KaBOB representations are predominantly currently modeled in OWL-EL [53], which can be computed in polynomial time, though we have yet to attempt to run a classifier over billions of triples. (The only representational construct outside of OWL-EL currently in KaBOB is union, which is used in the formal definition of the sequence abstraction classes as unions of base sequence types; however, they can be represented in parallel as superclasses of the base sequence types). There is also ongoing work by triplestore providers to perform inference and materialization faster and for more complicated inferences [45,54]. Thus far we have taken advantage of SPARQL 1.1 property paths to reason through transitive properties (*e.g.*, subClassOf*); the performance of doing so varies by triplestore. With some tuning to query ordering, AllegroGraph can navigate these paths in satisfactory time, with the slowest queries running on the order of minutes to tens of minutes on our hardware. Alternately, transitive properties could be materialized into the triplestore in order to get better performance. Given the way Virtuoso currently implements property paths, such a step would be necessary to query with multiple paths. Not all inferences can be accessed with property paths alone, *e.g.*, entailments from transitive properties or subclass hierarchies nested in OWL restrictions. We are interested in using OWL-EL reasoners such as ELK [55] to attempt to make these queries tractable. Other alternatives include using Datalog forward chainers to materialize the necessary triples, such as the Allegro Graph Materializer [56] or RDFox [57].

Querying OWL using only SPARQL can be tedious, but this is also improving with time. SPARQL 1.1 provides significant improvements over 1.0 via property paths. Other APIs for querying triplestores and interacting with OWL are also being developed by the community. We are interested in developing other APIs for common tasks and queries, or extending our existing domain-specific languages to alleviate some of the strain. The rule language for KaBOB, for example, makes available several macros to significantly reduce the number of boilerplate triples humans have to produce when interacting with record ICEs.

As reasoning and query capabilities continue to improve we are also interested in developing tools for maintaining and monitoring the quality of KaBOB. This will include research into systems that query for potentially erroneous assertions in KaBOB, trace their provenance, and report on the collective set of rules and source records used to create the incorrect assertions. Prior to a specific resolution for these problems being provided by human intervention these assertions could be blocked from generation in subsequent builds of KaBOB or filtered from an existing KaBOB instance.

### Related work

Related research on the semantic integration of data sources falls into two primary classes: automated integration investigated by the computer science community (typically database or AI researchers), and the more manual but domain-specific research conducted by the bioinformatics community.

### Automated database integration

The problem of integrating multiple databases has a history almost as old as relational databases themselves; Doan and Halevy [58] review this work and provide links to older reviews on this topic. Research in the database and artificial intelligence (AI) communities on automated data integration falls into two main categories: schema matching and data matching. Schema matching focuses on mapping the schema for one database to another, *e.g.*, figuring out that the “Surname” column of one database is the same as the “Last Name” column of another. Data matching is performed using actual field values, *e.g.*, finding that a record in database A has the value “Livingston” and database B also has the value “Livingston” for a given record, and so on for the other values of the records.

Work on automated matching is complicated by the fact that schemas often model data at different level of abstraction and there is not necessarily a one-to-one mapping across databases. For example, one schema might have separate fields for street address, city, and state, while another schema might represent all of that data in an integrated address field. These complex matches can be difficult to identify, as increasing the number of fields that can be combined along with the ways in which they can be combined can result in a combinatorial explosion. When it comes to tuple matching there is the question of whether or not two nearly identical tuples represent duplicate or different data. There has also been growing evidence that there may not be one universally correct match, but rather that mappings are application-dependent [58]. Domain ontologies are also being used as a backbone for mapping multiple database schemas together [59]. KaBOB maps representations to ontologies allowing for multiple levels of abstraction to be represented simultaneously. These mappings are also currently produced manually, allowing knowledge engineers to use all available background knowledge (including database documentation) to generate the matches to the OBOs.

In the development of KaBOB we have opted for a manually built rule-based approach to schema matching, converting the matched data into a common model grounded in prominent biomedical ontologies. While our approach is potentially slower than automated approaches, there are a finite number of rules to be produced. Future work could involve exploring the use of automated mapping techniques or human-computer hybrid techniques to accelerate the process. However, the primary bottleneck is still creating the target knowledge representation for a new class of biomedical information; after a new such representation is constructed, a second source of the same type of information can be more easily built, copying from the first.

Doan and Halevy [58] state the core problem of semantic integration is identifying if any two elements refer to the same real-world concept. In KaBOB we explicitly model these mappings in terms of identifier sets and other iao:denotes links, as well as tracking the provenance of other concepts through declarative rules. Provenance and explanation of matches is not always provided by automated database integration systems, although it is also being researched by the database community [60].

### Biomedical database integration

Interchange languages, such as BioPAX [61], GAF2 [62], and PSI MIF [63] provide a common way to represent data and are playing a growing role in the biomedical data ecosystem for sharing data. These data formats have done much to increase the level of data sharing in the community; however they are generally domain- or task-specific. Larger database integration projects also exist, such as BioMart [64] which provides the ability to query across multiple biomedical data sources. While this provides a common interface, queries are still required to know which sources they wish to query and how the data is organized in those individual sources. Much work has gone into producing database integrations with various configurations and goals; Louie *et al.* [65] provide a review-level discussion of several existing biomedical data integration systems. The remainder of our discussion will focus on ontology-based integration of biomedical data, as this is most related to KaBOB.

Semantic integration and querying of multiple sources of biomedical data dates back at least to Tambis [66], which attempted to answer queries without the user having to know which data sources were necessary. More recently, there has been a push to integrate biomedical data with the Semantic Web [67,68]. This includes work in building canonicalized and stable URIs [69,70] as well as work on publishing and disseminating data and recording their provenance [71,72].

Bio2RDF [73] is the most prominent project for providing access to RDF versions of many existing biomedical data sources. It provides canonical identifiers, and although it does not attempt to explicitly assert sets of identifiers that point to the same entities, it does provide access to identifier mappings; in this way it is primarily analogous to the ICE portions of KaBOB. Additionally, Bio2RDF does not attempt to align representations to a common biomedical model, thus making querying across data sources more difficult and convoluted in that query writers must navigate all relevant component database schemas themselves. Bio2RDF generates representations using source-specific models and is primarily focused on facilitating “mash-ups”. Given this focus, Bio2RDF has weaker constraints on its representation and sometimes conflates things that should be semantically distinct. For example, the URI for glutathione in Bio2RDF (http://bio2rdf.org/drugbank:DB00143) is explicitly asserted as rdf:typehttp://bio2rdf.org/drugbank_vocabulary:Drug and implicitly as foaf:Document (via a rdfs:domain constraint on an explicit void:inDataset assertion). These representational inconsistencies create a weak foundation if not real problems for research that wishes to build upon it for semantic reasoning purposes. While it may be possible to build KaBOB on top of something like Bio2RDF data, this remains an open question and entails risks. We believe our ICE representations provide a maximally general, and representationally sound, foundation for KaBOB.

Subsequent work points out that “querying Bio2RDF remains difficult due to the lack of uniformity in the representation of Bio2RDF datasets” [74]. Their work attempts to resolve this problem by aligning the source-specific schemas of Bio2RDF to the Semanticscience Integrated Ontology (SIO). They acknowledge that the SIO is limited in its coverage and needs to be extended, thus introducing a significant additional ontology development problem. The manual mappings done by Callahan *et al.* [74] require one-to-one relations between the source classes and properties in the Bio2RDF ontologies to those in the SIO, which they acknowledge are often imprecise matches to high-level concepts.

KaBOB makes very explicit the differences between database entities and biomedical concepts, unlike Bio2RDF. Its shared biomedical representations are grounded in prominent, actively maintained, large-scale Open Biomedical Ontologies. Compared to the SIO they have much greater coverage, greater consensus among the community, and are already used directly by many prominent curated databases, greatly simplifying the mapping problem. KaBOB also performs the translation from implicit database content to explicit biomedical representations using declaratively represented forward-chaining rules that are capable of dynamically constructing new entities. In contrast to approaches that only align ontology terms, this allows mismatches between the abstractions implicitly used for curation in the databases and those in the OBOs to be more easily overcome, while still recording provenance. When a rule (mapping) fails (perhaps due to changes in the underlying data), it is also easily detected as the output of the rule will produce zero triples, unlike mappings provided in hand-coded ontology files. Neurocommons [75] is another ontology-based knowledge base aggregating biomedical information from a range of sources. Precise identifiers for records that maintain the distinction between entities such as genes and gene records have been carefully created, and they are used for provenance of biomedical assertions, but, unlike KaBOB, the content of the records is not directly modeled. Record content that has not been mapped to a common biomedical model in Neurocommons is computationally inaccessible, and the mappings to the model are performed via procedural code. In KaBOB, record data is available for computational systems in the ICE portion of the knowledge base even if rules that map these data to biomedical representations have yet to be written or run. Furthermore, KaBOB’s declaratively represented rules can also function as provenance for concepts in the common biomedical model.

There have been several other approaches to semantic integration of biomedical data. Early work by Ruttenberg *et al.* [76] discussed the need for uniformly structured data across domains in order to advance translational research. They further discuss how the Semantic Web might provide a platform for such a taks. BioGateway [77] was an approach that aggregated a large quantity of data using a mixture of OBOs and custom ontologies. Like most earlier work it did not make a distinction between ICE and BIO content as does KaBOB nor does is integrate overlapping content. The work by Marshall *et al.* [78] also has similar goals to KaBOB; however it stops short in many key areas, leaving as opportunities for future development problems that KaBOB resolves, such as how to represent and integrate concepts representing information artifacts and how can they be used to provide provenance. It does solve other problems in ways that KaBOB has also adopted, such as normalizing the identifiers in source records. More recently work by Hoehndorf *et al.* [79] demonstrates how integrating data from multiple sources using OWL can support very complex querying. They integrate several types of data that KaBOB also provides; however their model forces the use of certain abstractions, for example, it is gene-centric mapping all data to genes, as opposed to supporting multiple parallel abstractions as does KaBOB. Their mappings are also done without making an ICE-BIO distinction and preserving the information entities as provenance. Finally they use equivalent class axioms to merge entities in the biomedical representations, while KaBOB resolves these mappings at an earlier stage with identifier mappings.

Other systems for indexing and querying federated sources of biomedical data have also been developed. ResourceIndex [80] indexes data sources and their records for search by using the NCBO Annotator [81] to identify the ontology concepts mentioned in them. ResourceIndex enriches its annotations using intra-ontology information, such as the transitivity of the subclass hierarchy; and inter-ontology information, such as mappings between ontologies. NIF [82] is a neuroscience-specific environment for indexing and querying Web pages, publications, and even databases. NIF provides the ability to use ontologies to query across data sources and even into databases by providing an environment where data providers can register their data and map it to the common ontologies, and where users can issues queries that are translated into ontology concepts and federated to all participating resources. Queries can be expanded to use a neighborhood of related ontology terms. Systems like ResourceIndex and NIF provide incredible querying power and specificity to users. However, these tools do not model the relationships between concepts present in records or documents, and they do not link or aggregate data across sources.

Existing commercial work on biomedical data integration includes Ingenuity Pathway Analysis [83], which identifies relevant molecular networks by integrating gene expression data, gene annotations, and manually curated data from literature. Other tools such as BioXM [84] provide a platform for data integration using a custom knowledge model and provide access for querying of these data. In addition to its reliance on large, prominent biomedical ontologies developed with significant community input for the structuring of its integrated data, KaBOB notably differs from these in being freely licensed.

## Conclusions

We presented five processes that when collectively applied provide solutions to seven key semantic data integration issues. We applied these processes to 18 large biomedical data sources to produce KaBOB (the Knowledge Base of Biomedicine), an integrated knowledge base of biomedical data representationally based in prominent, actively maintained Open Biomedical Ontologies, thus enabling queries of the underlying data in terms of biomedical concepts (*e.g.*, genes and gene products, interactions and processes) rather than features of source-specific data schemas or file formats. In KaBOB, identity is resolved through the representation of biomedical concepts that are referred to by sets of identifiers, making no preferential commitments to any identifier space. Declaratively represented forward-chaining rules take information that is variably represented in disparate independent underlying database models and generate representations in a common ontology-based biomedical model but also leave the underlying source data available for querying and provenance. These rules also function to track provenance and allow all transformations to be inspected and evaluated. Common biomedical abstractions are used to take into account the ambiguity of modeling within source data and to reflect this ambiguity in queries of these data. KaBOB resolves many of the issues that routinely plague biomedical researchers intending to work with data from multiple data sources and provides a platform for ongoing data integration and development and for formal reasoning over a wealth of integrated biomedical data.

### Availability of supporting data

We provide and maintain the open source code that achieves the steps described above, from downloading source files to running the rules. Usage agreements of some data sources prohibit redistribution so we cannot redistribute the complete set of triples. We welcome inquiries about specific collaborations. We intend to develop tools to expose parameterized questions for more general consumption as well.

Datasources library: https://github.com/UCDenver-ccp/datasource

KR library: https://github.com/drlivingston/kr

KaBOB library: https://github.com/drlivingston/kabob

## Additional files

Additional file 1: Appendix A. Current KaBOB Build Procedure. Appendix A describes the steps and their sequence used to build KaBOB in more detail than is depicted in Figure 1. Additional file 2: Appendix B. Example Rule. Appendix B provides an example rule in the KR rule language and a corresponding discussion. This is the actual rule used to convert from GO biological process annotations to biomedical concepts that is discussed in the paper and depicted in part in Figure 2. Additional file 3: Appendix C. Example Queries. Appendix C provides several examples of querying KaBOB’s integrated data using the common OBO-based biomedical model. Additional file 4: Appendix D. Error Detection. Appendix D provides more detailed examples of error detection queries with KaBOB. Additional file 5: Appendix E. DrugBank Identifier Mapping Errors. Appendix E provides more detail to one of the examples discussed in Appendix D.
