# Supplementary material for: KaBOB: ontology-based semantic integration of biomedical databases
Source: BMC Bioinformatics. 2015 Apr 23;16(1):126. doi: 10.1186/s12859-015-0559-3 (PMC4448321; doi:10.1186/s12859-015-0559-3)
Supplement: Additional file 1: Appendix A. — Current KaBOB Build Procedure. Appendix A describes the steps and their sequence used to build KaBOB in more detail than is depicted in Figure 1. [file 12859_2015_559_MOESM1_ESM.pdf]

Appendix A: Current KaBOB Build Procedure

KaBOB is currently built using the following 21 steps:

1. download ontology files
2. download database source files
3. rdf-ize database source files
4. load ontology files (from step 1)
5. run bio-to-ice rules (rules that generate ICE identifiers for each of the classes in the ontologies)
6. load RDF (from step 5)
7. load ICE RDF files (from step 3)
8. run id_typing rules
9. load RDF (from step 8)
10. run id_merging rules
11. load RDF (from step 10)
12. generate ID sets by computing a transitive closure of the mappings from step 10
13. load RDF (from step 12)
14. reify BIO entities for each ID set
15. load RDF (from step 14)
16. run rules for adding parent classed to entities (knowing that a class is a gene, protein etc. is needed for step 18)
17. load RDF (from step 16)
18. run rules for linking genes and gene products (connecting genes to gene products is needed for subsequent BIO rules since sources are curated at varying levels of detail)
19. load RDF (from step 18)
20. run remaining ICE to BIO rules
21. load RDF (from step 20)

At the end of this process is both a loaded instance of KaBOB and the source RDF files necessary to load another instance without having to run any of the generative processes (e.g., skipping steps 1,2,3,5,8,10,12,14,16,18,20). To integrate a new data source with KaBOB requires automating its download (step 2), writing a parser that will RDF-ize its contents(step 3), and then writing a series of rules as needed (not all sources require all rule steps) for: identifying and mapping source-specific identifiers (steps 8 and 10), adding entity-specific types, e.g., gene or gene products (step 16), entity linking rules necessary to create abstractions needed for the final BIO rules (step 18), and finally rules to generate additional BIO assertions (step 20). The level

of effort and number of rules varies from source to source. A large multi-faceted source like UniProt will need all of the steps. Whereas extracting only drug-gene interactions from DrugBank would require downloading(2), RDF-ization(3), identifying(8) and mapping(10) identifiers, and then a final rule to link drug to a gene or gene product (20). While the drug-gene relation rule certainly requires information generated in steps 16 and 18, in this particular case DrugBank does not add information to these steps and the knowledge engineer integrating it need not write any additional rules for those steps.
