# Supplementary material for: KaBOB: ontology-based semantic integration of biomedical databases
Source: BMC Bioinformatics. 2015 Apr 23;16(1):126. doi: 10.1186/s12859-015-0559-3 (PMC4448321; doi:10.1186/s12859-015-0559-3)
Supplement: Additional file 2: Appendix B. — Example Rule. Appendix B provides an example rule in the KR rule language and a corresponding discussion. This is the actual rule used to convert from GO biological process annotations to biomedical concepts that is discussed in the paper and depicted in part in Figure 2. [file 12859_2015_559_MOESM2_ESM.pdf]

Appendix B: Example Rule

Additional assertions in KaBOB are constructed using a series of declaratively represented forward-chaining rules. The rules have a body and a head that can share variables. The body assertions with variables are used to query the knowledge base. The variable bindings are then substituted into the assertions in the head of the rule and new triples are created. These triples are saved in compressed ntriple files, and then loaded into a KaBOB triplestore. The rules support references to new concepts in the head of the rule that are not referred to in the body. By default a unique new instances is reified for each of these unbound variables. The rule system also supports mechanisms for defining the new symbols in terms of other variable bindings. This allows multiple bindings for the same rule, or multiple rules to refer to the same entities without having to first look them up and see if they are already reified. It also allows the bindings for the rules to be processed in parallel as there in no dependencies or implied serialization in the result processing. The most common ways to reify new entities is to take an entity's name and add a prefix or suffix, or to use an SHA-1 hash of one or more of the other variable's bindings, creating a URI that is function of another set of URIs. The rules are represented using a domain-specific language (DSL) written using Clojure s-expressions. It is an extension of the pattern language provided in the open source KR Clojure library. The rules are applied using a straightforward implementation of a forward-chainer in Clojure. The rules could also be serialized to other formats. SWRL is an obvious potential target, however SWRL rules cannot have unbound variables in the head, thus blocking reification which is needed for many (although not all) rules. The rules could also be realized as SPARQL CONSTRUCT queries. This avenue has not been explored in great detail as when this project was started SPARQL 1.1 was still in its infancy and

access to the functions necessary to reify new entities was extremely limited. This can be re-investigated as future work, along with providing RIF export and import of rules. An example of a rule follows.

```
`{:name "goa-bp"
 :head
   ((?/bp rdfs/subClassOf ?/go) ;interaction

    (?/hr1 rdf/type           owl/Restriction)
    (?/hr1 owl/onProperty     obo/has_participant)
    (?/hr1 owl/someValuesFrom ?/bioentity)

    (?/bp rdfs/subClassOf ?/hr1))

 :body
   ((?/go   [rdfs/subClassOf *] obo/GO_0008150)
    (?/goid obo/IAO_0000219 ?/go)
    (?/fv0  obo/IAO_0000219 ?/goid)
    (?/fv0  kiao/hasTemplate
              iaogoa/GpAssociationGoaUniprotFileData_goIDDataField1)

    (?/record obo/has_part ?/fv0)
    (?/record obo/has_part ?/fv1)

    (?/fv1 kiao/hasTemplate
   iaogoa/GpAssociationGoaUniprotFileData_databaseObjectIDDataField1)
    (?/fv1 obo/IAO_0000219 ?/gp)
    (?/gp  obo/IAO_0000219 ?/bioentity)

    ;;filter out the negations
    (:optional
      ((?/record obo/has_part ?/qualfv)
       (?/qualfv kiao/hasTemplate
          iaogoa/GpAssociationGoaUniprotFileData_qualifierDataField1)
      (?/qualfv obo/IAO_0000219 ?/qualifier)))
    (:not (:regex ?/qualifier "^NOT" "i"))

    ;; it always a protein.
    ;; but if we wanted to go up to GorGP use the following
    ;; (_/gp obo/IAO_0000219 ?/bioentity))

    ;;(_/geneid   obo/IAO_0000219     ?/gene)
    ;;(?/gene     [rdfs/subClassOf *] ?/gorgporv)
    ;;(?/gorgporv rdf/type           kbio/GeneSpecificGorGPorVClass))
    )
 :reify ([?/bp {:ln (:sha-1 ?/go ?/hr1)
                :ns "kbio" :prefix "BP_"}]
         [?/hr1 {:ln (:restriction)
                :ns "kbio" :prefix "R_"}])

 :options {:magic-prefixes
          ;; tell AG this query is hand optimized, preserve order
          [["franzOption_clauseReorderer" "franz:identity"]]}
 }
```

The rule has three required parts (name, head, and body), and two optional parts (reify, and options). Since this is also Clojure syntax, semicolons comment out the remainder of the line. The body is queried for, and then the triples in the head are produced for each set of results. If there are unbound variables in the head they will be reified, the reify section will be consuted for additional description, for example, how to compute a consistent SHA-1 hash value. Finally the options section is for other non-standard information, for example, hints to the undlying triple stores on how to execute the query. These options will not affect queries to triplestores that are unaware of how to make use of them, they are merely submitted as additional namespaces in a SPARQL query in this case.
