# Supplementary material for: KaBOB: ontology-based semantic integration of biomedical databases
Source: BMC Bioinformatics. 2015 Apr 23;16(1):126. doi: 10.1186/s12859-015-0559-3 (PMC4448321; doi:10.1186/s12859-015-0559-3)
Supplement: Additional file 3: Appendix C. — Example Queries. Appendix C provides several examples of querying KaBOB’s integrated data using the common OBO-based biomedical model. [file 12859_2015_559_MOESM3_ESM.pdf]

Appendix C: Example Queries

KaBOB presents an integrated view of multiple sources of data using a common biomedical model. This model makes it possible to write a variety of queries that incorporate information from multiple sources without necessarily needing to know the specific contents of each source. For example, the following the query asks for what binds with the gene SEPT2 or any of its products.

```
PREFIX rdfs: <http://www.w3.org/2000/01/rdf-schema#>
PREFIX owl: <http://www.w3.org/2002/07/owl#>
PREFIX obo: <http://purl.obolibrary.org/obo/>
PREFIX kbio: <http://kabob.ucdenver.edu/bio/>
PREFIX iaohgnc: <http://kabob.ucdenver.edu/iao/hgnc/>

SELECT ?participant WHERE {

 iaohgnc:HGNC_SEPT2_ICE obo:IAO_0000219 ?gene . # SEPT2

 ## gene specific gene or gene product abstraction ?gorgporv
 ?gene   rdfs:subClassOf* ?gorgporv.
 ?gorgporv rdf:type       kbio:GeneSpecificGorGPorVClass .

 ## all gene products
 ?geneproduct rdfs:subClassOf* ?gorgporv.

 ## get processes they participate in
 ?gpparticipant owl:someValuesFrom ? geneproduct.
 ?gpparticipant owl:onProperty     obo:has_participant .
 ?interaction   rdfs:subClassOf    ?gpparticipant.

 ## look at only interactions
 ?interaction rdfs:subClassOf obo:MI_0000.         # interaction

 ## get all the other participants in those interactions
 ?interaction rdfs:subClassOf ?rparticipant.
 FILTER (?gpparticipant != ?rparticipant) .        # insure different
 ?rparticipant owl:onProperty     obo:has_participant .
 ?rparticipant owl:someValuesFrom ?participant.
}
```

This query retrieves other proteins as binding partners, including Polyubiquitin-C, which ultimately come from information in protein interaction databases, such as iRefWeb, as well as retrieving bindings for small molecules in ChEBI, including GTP. While the first results come directly from the modeling of iRefWeb in KaBOB the second come form a series of sources that when collectively integrated allow the query to be answered. First the rules in KaBOB create assertions from GO

Annotations, one of which in this case is an annotation from protein Septin-2 to GTP Binding (`obo:GO_0005525`) in the Gene Ontology. The cross-product definitions linking the GO to ChEBI define that term as something that `ro:has_input` (a `rdfs:subPropertyOf ro:has_participant`) some GTP (`obo:ChEBI_15996`). Thus with one straightforward biomedical query KaBOB can return results form a collection of integrated data sources, some of which, or at least the ways of combining them, might have been previously unknown to the author of the query.

Another example of a complex query across multiple data sources would be to ask, "what processes involve gene products that bind with insulin?" This can be asked of KaBOB with the following query.

```
PREFIX rdfs: <http://www.w3.org/2000/01/rdf-schema#>
PREFIX owl: <http://www.w3.org/2002/07/owl#>
PREFIX obo: <http://purl.obolibrary.org/obo/>
PREFIX kbio: <http://kabob.ucdenver.edu/bio/>
PREFIX iaouniprot: <http://kabob.ucdenver.edu/iao/uniprot/>

SELECT DISTINCT ?processName
WHERE {
 ## the human insulin protein
 iaouniprot:UNIPROT_P01308_ICE obo:IAO_0000219 ?insulin .

 ## processes that this protein participates in
 ?insParticipant owl:someValuesFrom ?insulin .
 ?insParticipant owl:onProperty     obo:has_participant .
 ?interaction    rdfs:subClassOf    ?insParticipant.

 ## look at only interactions
 ?interaction rdfs:subClassOf obo:MI_0000.         # interaction

 ## get all the other participants in those interactions
 ?interaction rdfs:subClassOf ?rparticipant.
 FILTER (?insParticipant != ?rparticipant) .       # insure different
 ?rparticipant owl:onProperty     obo:has_participant .
 ?rparticipant owl:someValuesFrom ?participant.

 ## gene specific gene or gene product abstraction ?gorgporv
 ?participant rdfs:subClassOf* ?gorgporv.
 ?gorgporv    rdf:type         kbio:GeneSpecificGorGPorVClass .

 ## all gene products
 ?geneproduct rdfs:subClassOf* ?gorgporv.

 ## get processes they participate in
 ?gpparticipant owl:someValuesFrom ?geneproduct.
 ?gpparticipant owl:onProperty     obo:has_participant .
 ?process       rdfs:subClassOf    ?gpparticipant.

```

```
 ## get the labeled names for those processes or parents
 ?process       rdfs:subClassOf    ?parentProcess.
 ?parentProcess rdfs:label ?processName .
}
```

A sample of 20 results for this query include a wide variety of processes.

```
"cellular response to L-ascorbic acid"
"forebrain development"
"positive regulation of steroid hormone biosynthetic process"
"transport"
"carbohydrate metabolic process"
"regulation of gene expression"
"peptidyl-tyrosine phosphorylation"
"glucose transport"
"negative regulation of G1/S transition of mitotic cell cycle"
"insulin-like growth factor receptor signaling pathway"
"positive regulation of protein phosphorylation"
"positive regulation of luteinizing hormone secretion"
"positive regulation of insulin receptor signaling pathway"
"extracellular matrix organization"
"endocytosis"
"autophagic vacuole assembly"
"small molecule metabolic process"
"phosphatidylinositol-mediated signaling"
"regulation of JNK cascade"
"signal transduction"
```

There are no logical constrainsts on the ways queries can be assembled. It is really up to the needs and imagination of a given querier as to how they assemble the various building blocks that KaBOB provides, and the constraints between them. The following is a more open-ended question, asking “find six pairs of proteins that have been localized to the same cellular location and that are known to interact.”

```
PREFIX rdfs: <http://www.w3.org/2000/01/rdf-schema#>
PREFIX owl: <http://www.w3.org/2002/07/owl#>
PREFIX obo: <http://purl.obolibrary.org/obo/>
PREFIX kbio: <http://kabob.ucdenver.edu/bio/>

SELECT DISTINCT ?gp1 ?gp2 ?locationName
WHERE {

 ## get localization events
 ?loc1 rdfs:subClassOf obo:GO_0051179 . #localization

 ## get the entity localized
 ?loc1 rdfs:subClassOf ?gp1Loc .
 ?gp1Loc owl:onProperty kro:results_in_localization_of .
 ?gp1Loc owl:someValuesFrom ?gp1 .

 ## get the location
 ?loc1 rdfs:subClassOf ?to .
 ?to owl:onProperty kro:results_in_localization_to .
 ?to owl:someValuesFrom ?location .
```

```
  ## find a second distinct localization to the same place
  ?loc2 rdfs:subClassOf ?to .
  FILTER (?loc1 != ?loc2) .        # insure different

  ## get the potentially “co-localized” entity
  ?loc2 rdfs:subClassOf obo:GO_0051179 . #localization
  ?loc2 rdfs:subClassOf ?gp2Loc .
  ?gp2Loc owl:someValuesFrom ?gp2 .
  ?gp2Loc owl:onProperty kro:results_in_localization_of .

  ## events the first entity participates in
  ?gp1Participant owl:someValuesFrom ?gp1 .
  ?gp1Participant owl:onProperty     obo:has_participant .
  ?interaction    rdfs:subClassOf    ?gp1Participant .

  ## look at only interactions
  ?interaction rdfs:subClassOf obo:MI_0000.         # interaction

  ## get all the other participants in those interactions
  ?interaction rdfs:subClassOf ?gp2Participant.
  FILTER (?gp1Participant != ?gp2Participant) .     # insure different

  ## make sure one is the second entity
  ?gp2Participant owl:onProperty     obo:has_participant .
  ?gp2Participant owl:someValuesFrom ?gp2.

  ## name of the location
  ?location rdfs:label ?locationName .
 }  LIMIT 6
```

Results

```
gp1 gp2 locationName
kbio:BIO_670debb05550269e5adf2a119a89f2a1
      kbio:BIO_44e82554526174a6eeacd1a7a6178fd9 "centriole"
kbio:BIO_a0759372c6bc720a973fec9e9f0c7d01
      kbio:BIO_2831ebac6d574c3a3a5c0df0be859de9 "COP9 signalosome"
kbio:BIO_82833770d0f5d98f8288061b647b7df7
      kbio:BIO_c0ad0de01140a27b99193fad4ea5792f "cytoplasm"
kbio:BIO_5bcbdafdbd53557f269c0b1b3967c33f
      kbio:BIO_56b655f5b336a3351ddb0aec99e1ccab "plasma membrane"
kbio:BIO_30d96024f2c92179e84f8e51100ea0f1
      kbio:BIO_f909f82a9f624e93ab7dd8ee44ea66d9 "nucleus"
kbio:BIO_703447445aeb3be0232764c81c022559
      kbio:BIO_67e86d637d474b346d6f8115a3a674f6 "proton-transporting
two-sector ATPase complex, catalytic domain"
```
