# Supplementary material for: KaBOB: ontology-based semantic integration of biomedical databases
Source: BMC Bioinformatics. 2015 Apr 23;16(1):126. doi: 10.1186/s12859-015-0559-3 (PMC4448321; doi:10.1186/s12859-015-0559-3)
Supplement: Additional file 4: Appendix D. — Error Detection. Appendix D provides more detailed examples of error detection queries with KaBOB. [file 12859_2015_559_MOESM4_ESM.pdf]

Appendix D: Error Detection

KaBOB's integrated biological model and its commitment to data provenance provide a unique opportunity for error detection when compared to other data integration systems. Errors can be detected by querying for concepts or links between concepts that should not exist. Once identified their provenance can be traced, and a solution can be identified. This capability is a critical aspect to the rule writing process and has been successfully used to identify and repair logic errors in rules. Errors in the source data can also be detected. In these cases it is often possible to adjust relevant rules can to correct for these problems. More rare are errors in a file parser causing the generation of erroneous RDF.

Disjoint Types

One straightforward thing to query for is to ensure that biomedical concepts do not exist that are of disjoint types. This can be accomplished with some targeted SPARQL queries, however, even more leverage could be gained here from the use of OWL reasoners, which is a source of future work. Things that can be queried for include ensuring entities are distinct from processes and that DNA and proteins are distinct from each other, which is shown in the following query:

```
PREFIX rdfs: <http://www.w3.org/2000/01/rdf-schema#>
PREFIX obo: <http://purl.obolibrary.org/obo/>

SELECT ?bio WHERE {

 ?bio rdfs:subClassOf* obo:SO_0000352 .  #DNA
 ?bio rdfs:subClassOf* obo:CHEBI_36080 . #protein
}
```

Identifier Collapse

Since KaBOB resolves identity across data sources by clustering sets of identifiers, it is important to detect if a set of identifiers has accidentally grown too large, causing what should be several distinct biomedical entities to collapse into a single identifier. This can be hard to detect prior to the identifier sets being generated. The ability to

inspect the consequences of these identifier mappings is an important advantage provided by KaBOB, as opposed to systems that require query writers to manually transverse these mappings themselves (as is the case with the current state of the art in biomedical pipeline building, or when using a system such as Bio2RDF).

One mode of identifier collapse is when a data source does not uniquely map to identifiers in another source, as we detected in DrugBank. These erroneous mappings resulted in 300 errors where two or more DrugBank identifiers were associated with the same external identifier. Since a transitive closure is computed over identifier mappings it is also possible to detect distant interactions not otherwise noticeable. For example, if there are mappings from source A to source B, B to source C, and then C to A, it is possible that the mappings do not agree and instead of tight loops of agreement being formed there are disagreements that when transitively closed cause too many identifiers to be merged. If you only looked at all the mappings in and out of a source those disagreements would not be seen.

The first naïve approach to detecting collapse is to look for sets of identifiers that include multiple identifiers from the same data source. However, since many data sources have redundant and deprecated identifiers, for example UniProt, this will not universally hold. Instead we look at the primary identifier fields for many of the data sources. Since these should all point to distinct biomedical entities, we can check for sets that contain two primary identifiers from UniProt, or from DugBank, etc. An example of this kind of error checking query looks like the following:

```
PREFIX rdfs: <http://www.w3.org/2000/01/rdf-schema#>
PREFIX obo: <http://purl.obolibrary.org/obo/>
PREFIX kiao: <http://kabob.ucdenver.edu/iao/>
PREFIX iaodrugbank: <http://kabob.ucdenver.edu/iao/drugbank/>

SELECT ?dbId1 ?dbId2 ?bio WHERE {

#look for the first DrugBank ID - primary ID field
?fv1 kiao:hasTemplate
      iaodrugbank:DrugBankDrugRecord_drugBankIdDataField1 .
```

```
?fv1 obo:IAO_0000219 ?dbId1 . #denotes

 ?dbId1 obo:IAO_0000219 ?bio . #denotes
 ?dbId2 obo:IAO_0000219 ?bio . #denotes

 FILTER (?dbId1 != ?dbId2) #make sure they are different

#look for the second DrugBank ID - primary ID field
 ?fv2 obo:IAO_0000219 ?dbId2 . #denotes
 ?fv2 kiao:hasTemplate
      iaodrugbank:DrugBankDrugRecord_drugBankIdDataField1 .
}
```

From this query we were initially able to detect identifier collapse. When we traced back the provenance we identified that DrugBank was non-uniquely mapping to external identifiers. These were being extracted using this rule:

```
`{:name "drugbank-drug-exact-mapping-assertion"
  :head ((?/dbice skos/exactMatch ?/otherice))
  :body
  ((?/fv0 kiao/hasTemplate
          iaodrugbank/DrugBankDrugRecord_drugBankIdDataField1)
   (?/fv0 obo/IAO_0000219  ?/dbice)
   (?/record obo/has_part  ?/fv0)

   (?/record obo/has_part  ?/externalfv)
   (?/externalfv kiao/hasTemplate
        iaodrugbank/DrugBankDrugRecord_externalIdentifiersDataField1)
   (?/externalfv obo/IAO_0000219  ?/otherice))
}
```

That rule was updated to remove the unreliable identifiers from consideration and KaBOB was able to be regenerated without these entities collapsing. The updated rule looks like the following:

```
`{:name "drugbank-drug-exact-mapping-assertion"
  :head ((?/dbice skos/exactMatch ?/otherice))
  :body
  ((?/fv0 kiao/hasTemplate
          iaodrugbank/DrugBankDrugRecord_drugBankIdDataField1)
   (?/fv0 obo/IAO_0000219  ?/dbice)
   (?/record obo/has_part  ?/fv0)

   (?/record obo/has_part  ?/externalfv)
   (?/externalfv kiao/hasTemplate
        iaodrugbank/DrugBankDrugRecord_externalIdentifiersDataField1)
   (?/externalfv obo/IAO_0000219  ?/otherice)

   ;;check to see if that fv is in another record
   (:optional ((?/record2 obo/has_part  ?/externalfv)
               (:not (= ?/record2 ?/record))))
   (:not (:bound ?/record2)))
}
```

A query to get a list of the detectable mis-mapped identifiers and its results is in Appendix E.

Another form of identifier collapse we have seen was due to the RDF file parsers accidentally truncating RefSeq identifiers. In this case all RefSeq identifiers were accidentally written as only their first few digits. This caused the majority of the entire human proteome to collapse to a single biomedical entity in KaBOB. This was immediately detected and then resolved.

Taxon Disagreement

Since we have taxon information for all the species-specific data in KaBOB, errors in the biomedical model can also be detected by looking for when entities from differing species are connected in inappropriate ways. The first most obvious thing to check for is a single biomedical entity being part of two disjoint taxa. Note that it is perfectly acceptable for a biomedical concept to have multiple taxon assertions, for example all the parent taxon classes are necessarily entailed by a child assertion, *e.g.*, all Homo sapiens (NCBI Taxon 9606) are also members of Mammalia (NCBI Taxon 40674). A query for an entity in two disjoint taxa is the following:

```
PREFIX rdfs: <http://www.w3.org/2000/01/rdf-schema#>
PREFIX owl: <http://www.w3.org/2002/07/owl#>
PREFIX obo: <http://purl.obolibrary.org/obo/>

SELECT ?dna ?tax1 ?tax2
WHERE {
  ?dna rdfs:subClassOf obo:SO_0000352 .              #DNA
  ?dna rdfs:subClassOf ?taxonrestriction1 .
  ?taxonrestriction1 owl:onProperty obo:RO_0002162 . #in_taxon
  ?taxonrestriction1 owl:someValuesFrom ?tax1 .

  ?dna rdfs:subClassOf ?taxonrestriction2 .
  ?taxonrestriction2 owl:onProperty obo:RO_0002162 . #in_taxon
  ?taxonrestriction2 owl:someValuesFrom ?tax2 .

  FILTER (?tax1 != ?tax2) .
  FILTER NOT EXISTS { ?tax1 rdfs:subClassOf* ?tax2 . } .
  FILTER NOT EXISTS { ?tax2 rdfs:subClassOf* ?tax1 . } .
}
```

The source data that KaBOB is derrived from is quite consistent in this regard and only six errors are propigated into KaBOB from erroneous data. These include one protein that is confused between mouse and rat, and five proteins that are simultaneously in two sibling yeast strains. The following are those results.

```
obo:NCBITaxon_10090 obo:NCBITaxon_10116 (mouse rat)
  kbio:BIO_e5838794206c4357c4fc0887ff9ef8f4
obo:NCBITaxon_10090 obo:NCBITaxon_10116 (yeast)
  kbio:BIO_c8742d154afdfecae05bbf42544456f8
  kbio:BIO_a7f26ae6f4a59610e55d080a894fb606
  kbio:BIO_4998abaafcf5a986229945dbfaef54f1
  kbio:BIO_19c1adaabcd380dece3abeca9b4cde54
```

Entities also stand in relationship to each other, for example, gene products (*e.g.*, proteins) are coded for by genes. Taxon information can also be leveraged here to look for misalignments by checking for a gene product from one taxa having a gene from a disjoint taxa as its template. A query that looks for that is the following:

```
PREFIX rdfs: <http://www.w3.org/2000/01/rdf-schema#>
PREFIX owl: <http://www.w3.org/2002/07/owl#>
PREFIX obo: <http://purl.obolibrary.org/obo/>

#retrive dna, gene-product, dna taxa, gene-product taxa
SELECT ?dna ?gptaxon2 ?tax1 ?tax2
WHERE {
  #DNA/gene in a taxa
  ?dna rdfs:subClassOf obo:SO_0000352 .             #DNA
  ?dna rdfs:subClassOf ?taxonrestriction1 .
  ?taxonrestriction1 owl:onProperty obo:RO_0002162 . #in_taxon
  ?taxonrestriction1 owl:someValuesFrom ?tax1 .

  #get the gene or gene product abstraction for that gene
  ?dna rdfs:subClassOf* ?gorgporv .
  ?gorgporv rdf:type kbio:GeneSpecificGorGPorVClass .

  #look at all the taxa of the member gene products
  ?gptaxon2 rdfs:subClassOf* ?gorgporv .
  ?gptaxon2 rdfs:subClassOf ?taxonrestriction2 .
  ?taxonrestriction2 owl:onProperty obo:RO_0002162 . #in_taxon
  ?taxonrestriction2 owl:someValuesFrom ?tax2 .

  #filter out those that are allowed
  FILTER (?tax1 != ?tax2) .
  FILTER NOT EXISTS { ?tax1 rdfs:subClassOf* ?tax2 . } .
  FILTER NOT EXISTS { ?tax2 rdfs:subClassOf* ?tax1 . } .
}
```
