# Supplementary material for: KaBOB: ontology-based semantic integration of biomedical databases
Source: BMC Bioinformatics. 2015 Apr 23;16(1):126. doi: 10.1186/s12859-015-0559-3 (PMC4448321; doi:10.1186/s12859-015-0559-3)
Supplement: Additional file 5: Appendix E. — DrugBank Identifier Mapping Errors. Appendix E provides more detail to one of the examples discussed in Appendix D. [file 12859_2015_559_MOESM5_ESM.pdf]

Appendix E: DrugBank Identifier Mapping Errors

The following query and results identifies errors in the mappings to external identifiers in DrugBank. See Appendix D for a more detailed discussion.

Query:

```
PREFIX obo: <http://purl.obolibrary.org/obo/>
PREFIX kiao: <http://kabob.ucdenver.edu/iao/>
PREFIX iaodrugbank: <http://kabob.ucdenver.edu/iao/drugbank/>

SELECT ?dbId1 ?dbId2 ?otherice WHERE {

  #look for the first DrugBank ID - primary ID field
  ?fv1 kiao:hasTemplate
       iaodrugbank:DrugBankDrugRecord_drugBankIdDataField1 .
  ?fv1 obo:IAO_0000219 ?dbId1 . #denotes

  #get the record for that field value
  ?record obo:has_part  ?fv1 .

  #get the mappings to external IDs in that record
  ?record obo:has_part  ?externalfv .
  ?externalfv kiao:hasTemplate
       iaodrugbank:DrugBankDrugRecord_externalIdentifiersDataField1 .
  ?externalfv obo:IAO_0000219  ?otherice .

  #find another record with that same external mapping
  ?record2 obo:has_part  ?externalfv .
  FILTER (?record != ?record2) .

  ## find the other primary ID in the second record
  ?record2 obo:has_part  ?fv2 .
  ?fv2 kiao:hasTemplate
       iaodrugbank:DrugBankDrugRecord_drugBankIdDataField1 .
  ?fv2 obo:IAO_0000219 ?dbId2 . #denotes
}
```

Results:

The result URIs have been post-processed to remove namespaces and other common parts such as “`_ICE`” to make the results easier to read.

```
dbId1 dbId2 otherice
"DB00002"   "DB00043"   "GENBANK_J00228"
"DB00002"   "DB00051"   "GENBANK_J00228"
"DB00002"   "DB00065"   "GENBANK_J00228"
"DB00002"   "DB00072"   "GENBANK_J00228"
"DB00002"   "DB00073"   "GENBANK_J00228"
"DB00002"   "DB00074"   "GENBANK_J00228"
"DB00002"   "DB00081"   "GENBANK_J00228"
"DB00002"   "DB00111"   "GENBANK_J00228"
"DB00006"   "DB02351"   "DPD_2246533"
"DB00008"   "DB00011"   "GENBANK_J00207"
"DB00008"   "DB00011"   "UNIPROT_P01563"
"DB00008"   "DB00022"   "UNIPROT_P01563"
```

51	"DB00008"	"DB00034"	"GENBANK_J00207"
52	"DB00008"	"DB00034"	"UNIPROT_P01563"
53	"DB00008"	"DB00069"	"GENBANK_J00207"
54	"DB00008"	"DB00105"	"UNIPROT_P01563"
55	"DB00009"	"DB00015"	"GENBANK_L00153"
56	"DB00009"	"DB00015"	"UNIPROT_P00750"
57	"DB00009"	"DB00029"	"GENBANK_L00153"
58	"DB00009"	"DB00029"	"UNIPROT_P00750"
59	"DB00009"	"DB00031"	"GENBANK_L00153"
60	"DB00009"	"DB00031"	"UNIPROT_P00750"
61	"DB00011"	"DB00022"	"UNIPROT_P01563"
62	"DB00011"	"DB00034"	"GENBANK_J00207"
63	"DB00011"	"DB00034"	"UNIPROT_P01563"
64	"DB00011"	"DB00069"	"GENBANK_J00207"
65	"DB00011"	"DB00105"	"UNIPROT_P01563"
66	"DB00012"	"DB00016"	"EMBL_X02158"
67	"DB00012"	"DB00016"	"UNIPROT_P01588"
68	"DB00015"	"DB00029"	"GENBANK_L00153"
69	"DB00015"	"DB00029"	"UNIPROT_P00750"
70	"DB00015"	"DB00031"	"GENBANK_L00153"
71	"DB00015"	"DB00031"	"UNIPROT_P00750"
72	"DB00019"	"DB00099"	"EMBL_X03438"
73	"DB00019"	"DB00099"	"UNIPROT_P09919"
74	"DB00022"	"DB00034"	"UNIPROT_P01563"
75	"DB00022"	"DB00105"	"UNIPROT_P01563"
76	"DB00023"	"DB00059"	"GENBANK_U00096"
77	"DB00023"	"DB00059"	"UNIPROT_P37595"
78	"DB00024"	"DB00066"	"GENBANK_M16647"
79	"DB00024"	"DB00066"	"UNIPROT_P01225"
80	"DB00024"	"DB00094"	"GENBANK_M16647"
81	"DB00024"	"DB00094"	"UNIPROT_P01225"
82	"DB00029"	"DB00031"	"GENBANK_L00153"
83	"DB00029"	"DB00031"	"UNIPROT_P00750"
84	"DB00030"	"DB00071"	"GENBANK_AY137503"
85	"DB00030"	"DB00071"	"UNIPROT_Q8HXV2"
86	"DB00032"	"DB00044"	"EMBL_X00264"
87	"DB00032"	"DB00044"	"UNIPROT_P01229"
88	"DB00034"	"DB00069"	"GENBANK_J00207"
89	"DB00034"	"DB00105"	"UNIPROT_P01563"
90	"DB00043"	"DB00051"	"GENBANK_J00228"
91	"DB00043"	"DB00051"	"UNIPROT_P01857"
92	"DB00043"	"DB00065"	"GENBANK_J00228"
93	"DB00043"	"DB00065"	"UNIPROT_P01857"
94	"DB00043"	"DB00072"	"GENBANK_J00228"
95	"DB00043"	"DB00072"	"UNIPROT_P01857"
96	"DB00043"	"DB00073"	"GENBANK_J00228"
97	"DB00043"	"DB00073"	"UNIPROT_P01857"
98	"DB00043"	"DB00074"	"GENBANK_J00228"
99	"DB00043"	"DB00074"	"UNIPROT_P01857"
100	"DB00043"	"DB00081"	"GENBANK_J00228"
101	"DB00043"	"DB00081"	"UNIPROT_P01857"
102	"DB00043"	"DB00111"	"GENBANK_J00228"
103	"DB00043"	"DB00111"	"UNIPROT_P01857"
104	"DB00051"	"DB00065"	"GENBANK_J00228"
105	"DB00051"	"DB00065"	"UNIPROT_P01857"
106	"DB00051"	"DB00072"	"GENBANK_J00228"
107	"DB00051"	"DB00072"	"UNIPROT_P01857"
108	"DB00051"	"DB00073"	"GENBANK_J00228"
109	"DB00051"	"DB00073"	"UNIPROT_P01857"
110	"DB00051"	"DB00074"	"GENBANK_J00228"
111	"DB00051"	"DB00074"	"UNIPROT_P01857"

112	"DB00051"	"DB00081"	"GENBANK_J00228"
113	"DB00051"	"DB00081"	"UNIPROT_P01857"
114	"DB00051"	"DB00111"	"GENBANK_J00228"
115	"DB00051"	"DB00111"	"UNIPROT_P01857"
116	"DB00052"	"DB00082"	"GENBANK_AF374232"
117	"DB00052"	"DB00082"	"UNIPROT_P58756"
118	"DB00053"	"DB00088"	"GENBANK_M16328"
119	"DB00053"	"DB00088"	"UNIPROT_P04062"
120	"DB00053"	"DB06720"	"UNIPROT_P04062"
121	"DB00060"	"DB00068"	"EMBL_V00534"
122	"DB00060"	"DB00068"	"UNIPROT_P01574"
123	"DB00062"	"DB00064"	"GENBANK_M12523"
124	"DB00062"	"DB00064"	"UNIPROT_P02768"
125	"DB00062"	"DB00096"	"GENBANK_M12523"
126	"DB00062"	"DB00096"	"UNIPROT_P02768"
127	"DB00064"	"DB00096"	"GENBANK_M12523"
128	"DB00064"	"DB00096"	"UNIPROT_P02768"
129	"DB00065"	"DB00072"	"GENBANK_J00228"
130	"DB00065"	"DB00072"	"UNIPROT_P01857"
131	"DB00065"	"DB00073"	"GENBANK_J00228"
132	"DB00065"	"DB00073"	"UNIPROT_P01857"
133	"DB00065"	"DB00074"	"GENBANK_J00228"
134	"DB00065"	"DB00074"	"UNIPROT_P01857"
135	"DB00065"	"DB00081"	"GENBANK_J00228"
136	"DB00065"	"DB00081"	"UNIPROT_P01857"
137	"DB00065"	"DB00111"	"GENBANK_J00228"
138	"DB00065"	"DB00111"	"UNIPROT_P01857"
139	"DB00066"	"DB00094"	"GENBANK_M16647"
140	"DB00066"	"DB00094"	"UNIPROT_P01225"
141	"DB00072"	"DB00073"	"GENBANK_J00228"
142	"DB00072"	"DB00073"	"UNIPROT_P01857"
143	"DB00072"	"DB00074"	"GENBANK_J00228"
144	"DB00072"	"DB00074"	"UNIPROT_P01857"
145	"DB00072"	"DB00081"	"GENBANK_J00228"
146	"DB00072"	"DB00081"	"UNIPROT_P01857"
147	"DB00072"	"DB00111"	"GENBANK_J00228"
148	"DB00072"	"DB00111"	"UNIPROT_P01857"
149	"DB00073"	"DB00074"	"GENBANK_J00228"
150	"DB00073"	"DB00074"	"UNIPROT_P01857"
151	"DB00073"	"DB00081"	"GENBANK_J00228"
152	"DB00073"	"DB00081"	"UNIPROT_P01857"
153	"DB00073"	"DB00111"	"GENBANK_J00228"
154	"DB00073"	"DB00111"	"UNIPROT_P01857"
155	"DB00074"	"DB00081"	"GENBANK_J00228"
156	"DB00074"	"DB00081"	"UNIPROT_P01857"
157	"DB00074"	"DB00111"	"GENBANK_J00228"
158	"DB00074"	"DB00111"	"UNIPROT_P01857"
159	"DB00081"	"DB00111"	"GENBANK_J00228"
160	"DB00081"	"DB00111"	"UNIPROT_P01857"
161	"DB00088"	"DB06720"	"UNIPROT_P04062"
162	"DB00088"	"DB08876"	"KEGG_D09675"
163	"DB00116"	"DB02031"	"KEGG_C00101"
164	"DB00123"	"DB03252"	"PUBCHEM_COMPOUND_5962"
165	"DB00130"	"DB02174"	"GUIDE_TO_PHARMACOLOGY_723"
166	"DB00130"	"DB02174"	"IUPHAR_723"
167	"DB00130"	"DB02174"	"PUBCHEM_COMPOUND_5961"
168	"DB00135"	"DB03839"	"PUBCHEM_COMPOUND_6057"
169	"DB00142"	"DB02517"	"GUIDE_TO_PHARMACOLOGY_1369"
170	"DB00142"	"DB02517"	"IUPHAR_1369"
171	"DB00142"	"DB02517"	"PUBCHEM_COMPOUND_33032"
172	"DB00150"	"DB03225"	"PUBCHEM_COMPOUND_6305"

173	"DB00151"	"DB03201"	"PUBCHEM_COMPOUND_5862"
174	"DB00160"	"DB01786"	"GUIDE_TO_PHARMACOLOGY_720"
175	"DB00160"	"DB01786"	"IUPHAR_720"
176	"DB00160"	"DB01786"	"PUBCHEM_COMPOUND_5950"
177	"DB00172"	"DB02853"	"GUIDE_TO_PHARMACOLOGY_3314"
178	"DB00172"	"DB02853"	"IUPHAR_3314"
179	"DB00172"	"DB02853"	"PUBCHEM_COMPOUND_145742"
180	"DB00182"	"DB01576"	"GUIDE_TO_PHARMACOLOGY_2147"
181	"DB00182"	"DB01576"	"IUPHAR_2147"
182	"DB00300"	"DB08819"	"PDB_TFO"
183	"DB00338"	"DB00736"	"GUIDE_TO_PHARMACOLOGY_4279"
184	"DB00338"	"DB00736"	"IUPHAR_4279"
185	"DB00382"	"DB03672"	"PDB_760"
186	"DB00407"	"DB01109"	"CHEMSPIDER_751"
187	"DB00407"	"DB01225"	"CHEMSPIDER_751"
188	"DB00407"	"DB01225"	"PUBCHEM_COMPOUND_772"
189	"DB00422"	"DB06701"	"PUBCHEM_COMPOUND_4158"
190	"DB00438"	"DB03530"	"PDB_CAZ"
191	"DB00452"	"DB00994"	"CHEMSPIDER_8075"
192	"DB00452"	"DB00994"	"PUBCHEM_COMPOUND_8378"
193	"DB00493"	"DB04181"	"PDB_CEF"
194	"DB00512"	"DB04529"	"PDB_DVV"
195	"DB00513"	"DB04134"	"PUBCHEM_COMPOUND_564"
196	"DB00570"	"DB02868"	"PDB_KAR"
197	"DB00592"	"DB03417"	"PDB_169"
198	"DB00624"	"DB02901"	"PDB_DHT"
199	"DB00676"	"DB02775"	"PUBCHEM_COMPOUND_2345"
200	"DB00688"	"DB01024"	"CHEBI_168396"
201	"DB00712"	"DB03753"	"PDB_FL2"
202	"DB00741"	"DB07886"	"CHEMSPIDER_5551"
203	"DB00741"	"DB07886"	"PUBCHEM_COMPOUND_5754"
204	"DB00813"	"DB08819"	"BINDING_DB_50008984"
205	"DB00813"	"DB08819"	"DPD_2240434"
206	"DB00825"	"DB00936"	"DPD_2241935"
207	"DB00879"	"DB08819"	"KEGG_C12599"
208	"DB00898"	"DB03045"	"PDB_168"
209	"DB00956"	"DB01551"	"KEGG_C08024"
210	"DB00984"	"DB08804"	"DPD_270687"
211	"DB01053"	"DB02968"	"PDB_PG1"
212	"DB01103"	"DB02240"	"PDB_QUM"
213	"DB01104"	"DB08567"	"CHEMSPIDER_61881"
214	"DB01104"	"DB08567"	"PUBCHEM_COMPOUND_68617"
215	"DB01109"	"DB01225"	"CHEMSPIDER_751"
216	"DB01128"	"DB02932"	"CHEMSPIDER_50614"
217	"DB01165"	"DB03034"	"PDB_XED"
218	"DB01275"	"DB01563"	"KEGG_D01302"
219	"DB01370"	"DB01378"	"DPD_13838"
220	"DB01608"	"DB01609"	"DPD_1926756"
221	"DB01646"	"DB01746"	"DPD_285528"
222	"DB01646"	"DB01786"	"DPD_285528"
223	"DB01646"	"DB02556"	"DPD_285528"
224	"DB01646"	"DB02853"	"DPD_285528"
225	"DB01655"	"DB02076"	"CHEMSPIDER_409"
226	"DB01663"	"DB03875"	"CHEMSPIDER_21594036"
227	"DB01663"	"DB03875"	"PUBCHEM_COMPOUND_23644225"
228	"DB01687"	"DB02061"	"CHEMSPIDER_288"
229	"DB01687"	"DB02061"	"PUBCHEM_COMPOUND_46936190"
230	"DB01687"	"DB02743"	"CHEMSPIDER_288"
231	"DB01687"	"DB02743"	"PUBCHEM_COMPOUND_46936190"
232	"DB01687"	"DB03323"	"PUBCHEM_COMPOUND_46936190"
233	"DB01688"	"DB01776"	"DPD_332984"

234	"DB01692"	"DB02184"	"CHEMSPIDER_17939"
235	"DB01692"	"DB02184"	"PUBCHEM_COMPOUND_446094"
236	"DB01692"	"DB04447"	"PUBCHEM_COMPOUND_446094"
237	"DB01697"	"DB03277"	"PUBCHEM_COMPOUND_46936192"
238	"DB01697"	"DB04248"	"PUBCHEM_COMPOUND_46936192"
239	"DB01709"	"DB03465"	"PUBCHEM_COMPOUND_439278"
240	"DB01725"	"DB06854"	"PUBCHEM_COMPOUND_5353305"
241	"DB01746"	"DB01786"	"DPD_285528"
242	"DB01746"	"DB02556"	"DPD_285528"
243	"DB01746"	"DB02853"	"DPD_285528"
244	"DB01756"	"DB03108"	"PUBCHEM_COMPOUND_446947"
245	"DB01786"	"DB02556"	"DPD_285528"
246	"DB01786"	"DB02853"	"DPD_285528"
247	"DB01822"	"DB02693"	"CHEMSPIDER_25754"
248	"DB01822"	"DB02693"	"PUBCHEM_COMPOUND_439407"
249	"DB01834"	"DB04187"	"PUBCHEM_COMPOUND_46936232"
250	"DB01839"	"DB02159"	"DPD_382302"
251	"DB01861"	"DB02421"	"PUBCHEM_COMPOUND_46936243"
252	"DB01861"	"DB03501"	"PUBCHEM_COMPOUND_46936243"
253	"DB01861"	"DB04355"	"PUBCHEM_COMPOUND_46936243"
254	"DB01870"	"DB03857"	"PUBCHEM_COMPOUND_445999"
255	"DB01872"	"DB04492"	"PUBCHEM_COMPOUND_440173"
256	"DB01894"	"DB02344"	"CHEMSPIDER_882"
257	"DB01894"	"DB02344"	"PUBCHEM_COMPOUND_36690637"
258	"DB01894"	"DB03721"	"PUBCHEM_COMPOUND_36690637"
259	"DB01894"	"DB04265"	"PUBCHEM_COMPOUND_36690637"
260	"DB01898"	"DB03810"	"PUBCHEM_COMPOUND_6604760"
261	"DB01954"	"DB03606"	"CHEMSPIDER_394978"
262	"DB01954"	"DB03606"	"PUBCHEM_COMPOUND_448055"
263	"DB01954"	"DB04149"	"PUBCHEM_COMPOUND_448055"
264	"DB01993"	"DB04467"	"PUBCHEM_COMPOUND_446862"
265	"DB02003"	"DB03871"	"PUBCHEM_COMPOUND_46936282"
266	"DB02007"	"DB02312"	"CHEMSPIDER_203"
267	"DB02007"	"DB02312"	"PUBCHEM_COMPOUND_46936284"
268	"DB02007"	"DB02900"	"PUBCHEM_COMPOUND_46936284"
269	"DB02007"	"DB04122"	"PUBCHEM_COMPOUND_46936284"
270	"DB02021"	"DB02101"	"CHEMSPIDER_3232"
271	"DB02021"	"DB02101"	"PUBCHEM_COMPOUND_449156"
272	"DB02038"	"DB03579"	"PUBCHEM_COMPOUND_445005"
273	"DB02038"	"DB03787"	"PUBCHEM_COMPOUND_445005"
274	"DB02053"	"DB02630"	"CHEMSPIDER_2133"
275	"DB02053"	"DB02630"	"PUBCHEM_COMPOUND_448027"
276	"DB02053"	"DB03745"	"PUBCHEM_COMPOUND_448027"
277	"DB02061"	"DB02743"	"CHEMSPIDER_288"
278	"DB02061"	"DB02743"	"PUBCHEM_COMPOUND_46936190"
279	"DB02061"	"DB03323"	"PUBCHEM_COMPOUND_46936190"
280	"DB02099"	"DB03985"	"CHEMSPIDER_16744174"
281	"DB02133"	"DB02863"	"PUBCHEM_COMPOUND_46936306"
282	"DB02135"	"DB02711"	"PUBCHEM_COMPOUND_657028"
283	"DB02171"	"DB02548"	"CHEMSPIDER_598"
284	"DB02171"	"DB02548"	"PUBCHEM_COMPOUND_14620017"
285	"DB02175"	"DB02201"	"KEGG_C00383"
286	"DB02184"	"DB04447"	"PUBCHEM_COMPOUND_446094"
287	"DB02218"	"DB03269"	"CHEMSPIDER_1672"
288	"DB02218"	"DB03269"	"PUBCHEM_COMPOUND_46936324"
289	"DB02228"	"DB04084"	"PUBCHEM_COMPOUND_5288324"
290	"DB02228"	"DB04282"	"PUBCHEM_COMPOUND_5288324"
291	"DB02228"	"DB04483"	"PUBCHEM_COMPOUND_5288324"
292	"DB02233"	"DB03412"	"PUBCHEM_COMPOUND_97725"
293	"DB02234"	"DB02539"	"CHEMSPIDER_4955"
294	"DB02234"	"DB02539"	"PUBCHEM_COMPOUND_5139"

295	"DB02235"	"DB02467"	"CHEMSPIDER_824"
296	"DB02235"	"DB02467"	"PUBCHEM_COMPOUND_9577091"
297	"DB02280"	"DB03357"	"PUBCHEM_COMPOUND_439616"
298	"DB02290"	"DB03022"	"PUBCHEM_COMPOUND_657024"
299	"DB02290"	"DB03812"	"PUBCHEM_COMPOUND_657024"
300	"DB02290"	"DB03973"	"PUBCHEM_COMPOUND_657024"
301	"DB02294"	"DB02471"	"CHEMSPIDER_4370"
302	"DB02294"	"DB02471"	"PUBCHEM_COMPOUND_46936338"
303	"DB02312"	"DB02900"	"PUBCHEM_COMPOUND_46936284"
304	"DB02312"	"DB04122"	"PUBCHEM_COMPOUND_46936284"
305	"DB02344"	"DB03721"	"PUBCHEM_COMPOUND_36690637"
306	"DB02344"	"DB04265"	"PUBCHEM_COMPOUND_36690637"
307	"DB02376"	"DB02525"	"CHEMSPIDER_3360"
308	"DB02376"	"DB02525"	"PUBCHEM_COMPOUND_10465168"
309	"DB02379"	"DB02687"	"CHEMSPIDER_201"
310	"DB02379"	"DB02687"	"PUBCHEM_COMPOUND_6992084"
311	"DB02379"	"DB02944"	"PUBCHEM_COMPOUND_6992084"
312	"DB02379"	"DB03989"	"PUBCHEM_COMPOUND_6992084"
313	"DB02387"	"DB03552"	"PUBCHEM_COMPOUND_6950578"
314	"DB02399"	"DB03815"	"PUBCHEM_COMPOUND_25032396"
315	"DB02421"	"DB03501"	"PUBCHEM_COMPOUND_46936243"
316	"DB02421"	"DB04355"	"PUBCHEM_COMPOUND_46936243"
317	"DB02456"	"DB03403"	"PUBCHEM_COMPOUND_5745914"
318	"DB02469"	"DB03439"	"PUBCHEM_COMPOUND_46936396"
319	"DB02480"	"DB04170"	"PUBCHEM_COMPOUND_448012"
320	"DB02556"	"DB02853"	"DPD_285528"
321	"DB02560"	"DB03607"	"PUBCHEM_COMPOUND_17753903"
322	"DB02577"	"DB03014"	"PUBCHEM_COMPOUND_4973"
323	"DB02590"	"DB04028"	"PUBCHEM_COMPOUND_46936431"
324	"DB02601"	"DB04291"	"PUBCHEM_COMPOUND_36143"
325	"DB02601"	"DB04308"	"PUBCHEM_COMPOUND_36143"
326	"DB02615"	"DB02715"	"CHEMSPIDER_10632305"
327	"DB02615"	"DB02715"	"PUBCHEM_COMPOUND_46936438"
328	"DB02630"	"DB03745"	"PUBCHEM_COMPOUND_448027"
329	"DB02666"	"DB04460"	"PUBCHEM_COMPOUND_46936448"
330	"DB02677"	"DB03290"	"PUBCHEM_COMPOUND_17754157"
331	"DB02682"	"DB03146"	"PUBCHEM_COMPOUND_46936452"
332	"DB02687"	"DB02944"	"PUBCHEM_COMPOUND_6992084"
333	"DB02687"	"DB03989"	"PUBCHEM_COMPOUND_6992084"
334	"DB02692"	"DB03918"	"PUBCHEM_COMPOUND_5702551"
335	"DB02714"	"DB02805"	"PUBCHEM_COMPOUND_46936464"
336	"DB02722"	"DB04303"	"PUBCHEM_COMPOUND_446874"
337	"DB02743"	"DB03323"	"PUBCHEM_COMPOUND_46936190"
338	"DB02765"	"DB03000"	"PUBCHEM_COMPOUND_445211"
339	"DB02788"	"DB04364"	"PUBCHEM_COMPOUND_447765"
340	"DB02807"	"DB03303"	"PUBCHEM_COMPOUND_21596768"
341	"DB02900"	"DB04122"	"PUBCHEM_COMPOUND_46936284"
342	"DB02934"	"DB03952"	"PUBCHEM_COMPOUND_46936531"
343	"DB02944"	"DB03989"	"PUBCHEM_COMPOUND_6992084"
344	"DB02976"	"DB03488"	"PUBCHEM_COMPOUND_46936545"
345	"DB03022"	"DB03812"	"PUBCHEM_COMPOUND_657024"
346	"DB03022"	"DB03973"	"PUBCHEM_COMPOUND_657024"
347	"DB03066"	"DB04398"	"GUIDE_TO_PHARMACOLOGY_2934"
348	"DB03066"	"DB04398"	"IUPHAR_2934"
349	"DB03066"	"DB04398"	"PUBCHEM_COMPOUND_61503"
350	"DB03109"	"DB03567"	"PUBCHEM_COMPOUND_440552"
351	"DB03109"	"DB03740"	"PUBCHEM_COMPOUND_440552"
352	"DB03188"	"DB03584"	"PUBCHEM_COMPOUND_46936599"
353	"DB03188"	"DB03599"	"PUBCHEM_COMPOUND_46936599"
354	"DB03194"	"DB03879"	"PUBCHEM_COMPOUND_46936603"
355	"DB03194"	"DB04091"	"PUBCHEM_COMPOUND_46936603"

"DB03206"	"DB03955"	"PUBCHEM_COMPOUND_44387838"
"DB03235"	"DB04073"	"PUBCHEM_COMPOUND_46936618"
"DB03271"	"DB04400"	"CHEMSPIDER_247"
"DB03271"	"DB04400"	"PUBCHEM_COMPOUND_5460203"
"DB03277"	"DB04248"	"PUBCHEM_COMPOUND_46936192"
"DB03401"	"DB03956"	"PUBCHEM_COMPOUND_10342059"
"DB03445"	"DB03834"	"PUBCHEM_COMPOUND_46936678"
"DB03492"	"DB04550"	"PUBCHEM_COMPOUND_46936698"
"DB03494"	"DB06855"	"CHEMSPIDER_20120240"
"DB03494"	"DB06855"	"PUBCHEM_COMPOUND_6102526"
"DB03501"	"DB04355"	"PUBCHEM_COMPOUND_46936243"
"DB03519"	"DB04185"	"PUBCHEM_COMPOUND_65098"
"DB03567"	"DB03740"	"PUBCHEM_COMPOUND_440552"
"DB03579"	"DB03787"	"PUBCHEM_COMPOUND_445005"
"DB03584"	"DB03599"	"PUBCHEM_COMPOUND_46936599"
"DB03606"	"DB04149"	"PUBCHEM_COMPOUND_448055"
"DB03721"	"DB04265"	"PUBCHEM_COMPOUND_36690637"
"DB03773"	"DB04319"	"PUBCHEM_COMPOUND_6713579"
"DB03812"	"DB03973"	"PUBCHEM_COMPOUND_657024"
"DB03863"	"DB04515"	"PUBCHEM_COMPOUND_46936810"
"DB03879"	"DB04091"	"PUBCHEM_COMPOUND_46936603"
"DB03883"	"DB04262"	"PUBCHEM_COMPOUND_46936818"
"DB04056"	"DB04166"	"PUBCHEM_COMPOUND_227"
"DB04084"	"DB04282"	"PUBCHEM_COMPOUND_5288324"
"DB04084"	"DB04483"	"PUBCHEM_COMPOUND_5288324"
"DB04127"	"DB04352"	"PUBCHEM_COMPOUND_46876838"
"DB04282"	"DB04483"	"PUBCHEM_COMPOUND_5288324"
"DB04291"	"DB04308"	"PUBCHEM_COMPOUND_36143"
"DB04313"	"DB04538"	"PUBCHEM_COMPOUND_5287600"
"DB05294"	"DB08764"	"CHEBI_49960"
"DB07914"	"DB07915"	"CHEMSPIDER_16188846"
"DB07914"	"DB07915"	"PUBCHEM_COMPOUND_5462192"
"DB08377"	"DB08411"	"CHEMSPIDER_4593437"
"DB08377"	"DB08411"	"PUBCHEM_COMPOUND_5496653"
"DB08648"	"DB08650"	"CHEMSPIDER_392988"
"DB08648"	"DB08650"	"PUBCHEM_COMPOUND_445314"
